# Computationally‐Guided Development of Sulfide Solid Electrolyte Powder Coatings for Enhanced Stability and Performance of Solid‐State Batteries

**DOI:** 10.1002/advs.202513191

**Published:** 2025-10-15

**Authors:** Aditya Sundar, Taewoo Kim, Francisco Lagunas, Anil U. Mane, Udochukwu D. Eze, Colton Ginter, Rajesh Pathak, Sanja Tepavcevic, Jeffrey W. Elam, Zachary D. Hood, Peter Zapol, Justin G. Connell

**Affiliations:** ^1^ Materials Science Division Argonne National Laboratory 9700 S Cass Ave. Lemont IL 60439 USA; ^2^ Applied Materials Division Argonne National Laboratory 9700 S Cass Ave. Lemont IL 60439 USA; ^3^ Pritzker School of Molecular Engineering The University of Chicago Chicago IL 60637 USA

**Keywords:** atomic layer deposition, interface design, lithium metal anodes, solid‐state batteries, sulfide solid electrolytes

## Abstract

Sulfide‐based solid‐state electrolytes (SSEs) such as Li_6_PS_5_Cl (LPSCl) are attractive Li^+^ superionic conductors for next generation solid state batteries whose narrow voltage window and environmental reactivity hinder widespread commercialization. These limitations can be mitigated by coating LPSCl powders with thin Al_2_O_3_ coatings via atomic layer deposition; however, design rules are needed to identify new coatings with further improved properties. Here, a density functional theory‐based screening protocol is developed to identify and experimentally demonstrate multiple new oxide coatings with multifaceted benefits. MgO coatings, in particular, improve the electronic conductivity, Li metal stability, interfacial resistance, and the critical current density of coated LPSCl powders. It is found that the ionic and electronic conductivity of reaction products formed at oxide interfaces with LPSCl and Li metal are the most predictive metrics for determining a viable coating. These results open a new frontier of research for improving the stability and performance of sulfide‐based SSEs.

## Graphical abstract

An integrated, computational‐experimental approach is developed to identify and demonstrate multiple new, oxide‐based coatings for Li_6_PS_5_Cl powders that improve ionic/electronic transport and (electro)chemical stability. This work identifies a new research frontier for exploring the myriad benefits of SSE powder coating to improve the stability, processability, and performance of sulfide‐based solid‐state batteries.

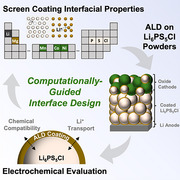

## 1 Introduction

Solid‐state batteries are a promising class of next‐generation energy storage technologies due to their increased energy density, wider operating temperature window, compact design, and enhanced capacities relative to current Li‐ion batteries.^[^ ^1^, ^2^, ^3^, ^4^, ^5^ ^]^ In particular, sulfide‐based solid‐state electrolytes (SSEs) have shown tremendous potential due to their promising superionic conduction, the abundance of their non‐toxic constituent elements, and their favorable mechanical properties.^[^ ^1^, ^6^ ^]^ Among sulfide‐based SSEs, argyrodite‐type materials with a cubic structure and Li_7‐y_PS_6‐y_X_y_ (LPSX; *X* = Cl, Br, I) composition are particularly attractive owing to their high ionic conductivities (>1 mS cm^−1^), wide bandgaps, and favorable processing properties.^[^ ^7^ ^]^ The small lattice constant and high anion site disorder of Li_6_PS_5_Cl (LPSCl) result in a more delocalized lithium density, leading to higher ionic conductivity values than some analogous halide‐substituted argyrodites.^[^ ^8^, ^9^, ^10^, ^11^, ^12^ ^]^

Design of all‐solid‐state batteries using LPSCl SSEs needs to address crucial challenges such as environmental instability and performance deterioration under operating conditions. Argyrodites also have well‐documented issues with environmental instability^[^ ^13^, ^14^, ^15^, ^16^, ^17^ ^]^ and reactivity with lithium metal,^[^ ^18^, ^19^, ^20^ ^]^ which leads to reduced interfacial contact,^[^ ^21^, ^22^ ^]^ structural instabilities and crack nucleation,^[^ ^23^ ^]^ volumetric shrinkage under oxidative decomposition and subsequent void formation at cathode interfaces^[^ ^24^ ^]^ – all of which are active areas of research throughout the sulfide solid electrolyte community. Beyond electrochemical stability, manufacturing of these materials in realistic industrial settings is also a critical issue, where environmental reactivity is a significant challenge to their processing at scale.

A common strategy for mitigating interfacial reactions of sulfide electrolytes with Li metal anodes and/or high‐voltage oxide cathode active materials is to apply coatings to these electrodes.^[^ ^25^, ^26^, ^27^, ^28^, ^29^, ^30^, ^31^ ^]^ This suppresses the formation of phosphates, sulfates, and sulfites, and enhances performance. To this end, DFT‐based predictive approaches have been used effectively to guide the development of coating materials to create stable interfaces in solid‐state batteries,^[^ ^32^, ^33^, ^34^, ^35^, ^36^, ^37^ ^]^ with a predominant focus on mitigating interfacial reactions by coating the cathode. Recent high‐throughput thermodynamic calculations identified several Li‐based oxide coatings that are stable against lithium lanthanum zirconium oxide (LLZO) SSEs and LiNi_x_Mn_y_Co_(1‐x‐y)_O_2_ (NMC) cathodes. In some cases, these coatings are also predicted to control undesirable reactions occurring during LLZO sintering.^[^ ^32^ ^]^ A similar thermodynamic modeling scheme revealed binary oxides, such as Al_2_O_3_, SiO_2_, and ZrO_2_, and lithium ternary oxides, such as Li_3_PO_4_, LiNbO_3_, and LiTaO_3_, as stable coatings on various cathodes.^[^ ^34^ ^]^ Polyanionic oxide coatings were also proposed for cathodes in solid‐state batteries using DFT‐based screening for phase stability, electrochemical and chemical stability, and ionic conductivity.^[^ ^35^ ^]^ Overall, guiding principles have been developed to accelerate the screening of interfacial coatings, and the validity of these screening criteria was demonstrated by establishing qualitative agreement between some of the predicted coating chemistries and previous experimental data.^[^ ^32^, ^33^, ^34^ ^]^ However, these screening procedures have focused almost exclusively on coatings intended to be applied to the cathode, and systematic evaluations of coating stability to interfaces throughout the entire solid‐state battery are generally lacking. Indeed, although studies on sulfide SSE‐cathode interfaces have considered a wide range of coating chemistries (e.g., oxides, phosphates, halides, etc…)^[^ ^35^ ^]^ as well as coating properties such as Li^+^ migration and bandgap,^[^ ^38^ ^]^ interfacial energy barriers,^[^ ^39^ ^]^ or interfacial resistance,^[^ ^40^ ^]^ a comprehensive examination of the properties of the decomposition products at these interfaces is also lacking.^[^ ^41^ ^]^

We have recently demonstrated an approach that differs from coating the cathode, where coating LPSCl powders with ≈1 nm Al_2_O_3_ via atomic layer deposition (ALD) improves the chemical stability of LPSCl, resulting in improved stability to oxidizing atmospheres^[^ ^42^ ^]^ and contact with Li metal.^[^ ^13^ ^]^ Moreover, the coated electrolyte powders also exhibit up to a two‐fold increase in ionic conductivity and reduced electronic conductivity as compared to uncoated powders, likely due to the formation of conductive ─Li─S─Li─O─Li─S─Li─ networks at the interface.^[^ ^13^ ^]^ The formation of such reaction products at solid‐solid interfaces is, in general, poorly understood, and developing systematic screening procedures to identify new, multifunctional coating chemistries that can improve ionic conductivity and materials stability without increasing electronic conductivity must account for the formation of reactive decomposition products that may also impact battery performance.^[^ ^32^, ^33^, ^34^, ^35^ ^]^

In this article, we demonstrate a combined computational‐experimental approach to discover multiple new coating chemistries that provide multifaceted benefits to the stability and performance of LPSCl SSEs. Our screening framework includes properties such as reaction energies at the coating||electrolyte, coating||cathode, and coating||anode interfaces, bandgap of the oxide in bulk and thin‐film forms, and ionic and electronic conductivity estimations for the interfacial reaction products. We first develop a two‐step, DFT‐based screening methodology to assess the stability of oxide‐based coatings on LPSCl across multiple metrics in order to determine the most predictive criteria for selecting candidate coating chemistries. High‐level screening in the first stage consists of identifying oxide coatings that have low reaction energies at various solid‐state interfaces and maintain a large bandgap, pointing to reduced electronic conductivity. Candidates identified using these criteria are then shortlisted, and detailed calculations are performed in the second stage to evaluate Li^+^ and electronic conductivity of reaction products formed in reactions computed in the first step. This comprehensive and systematic property screening procedure establishes stronger physical criteria to select coatings for experimental validation, rather than using reaction energies as the sole criterion.

Leveraging this screening methodology, we identify several candidate coating chemistries with a range of predicted reactivities (i.e., MgO, ZrO_2_, and ZnO), successfully employ ALD to deposit these coatings on LPSCl powders, and assess their resulting (electro)chemical properties relative to uncoated LPSCl. MgO and ZnO‐based coatings are shown to exhibit similar or enhanced ionic conductivity (up to 25% higher) relative to uncoated LPSCl, along with an order of magnitude decrease in electronic conductivity. In contrast, ZrO_2_‐based coatings, which are predicted to be among the most chemically stable oxides considered, exhibit an order of magnitude decrease in ionic conductivity and a factor of two increase in electronic conductivity. X‐ray photoelectron spectroscopy (XPS) analysis of coated materials indicates a mixture of reaction products predicted to form at interfaces between the oxide coating and both LPSCl and Li metal, suggesting that kinetics, rather than thermodynamics, govern interfacial stability. In contrast, the ionic and electronic conductivity of reaction products predicted to form at either interface align very well with the observed electrochemical properties of the coated materials. Specifically, Li‐conductive Li_2_O, lithiated oxides, and metal alloys are predicted and observed to form on MgO and ZnO‐coated materials, whereas Li‐insulating Li_6_Zr_2_O_7_ and Zr_3_O are predicted and observed to form on ZrO_2_‐coated materials. Taken together, our results indicate that the most predictive metric for selecting successful coating chemistries is the thermodynamic preference for forming Li alloys and Li‐conductive oxides at interfaces with LPSCl and Li metal. MgO coatings are further demonstrated to exhibit improved stability in contact with Li metal, reduced interfacial resistance, and improved critical current densities relative to uncoated LPSCl, demonstrating the power of this computational screening approach to identify new coating chemistries with multifaceted benefits to stability and performance. Overall, this work provides clear design rules for the discovery of coatings that yield exceptional performance of sulfide SSEs via tunable interfacial chemistry and transport properties.

## 2 Results

### 2.1 Thermodynamic Stability of Solid‐Solid Interfaces

**Figure 1:**
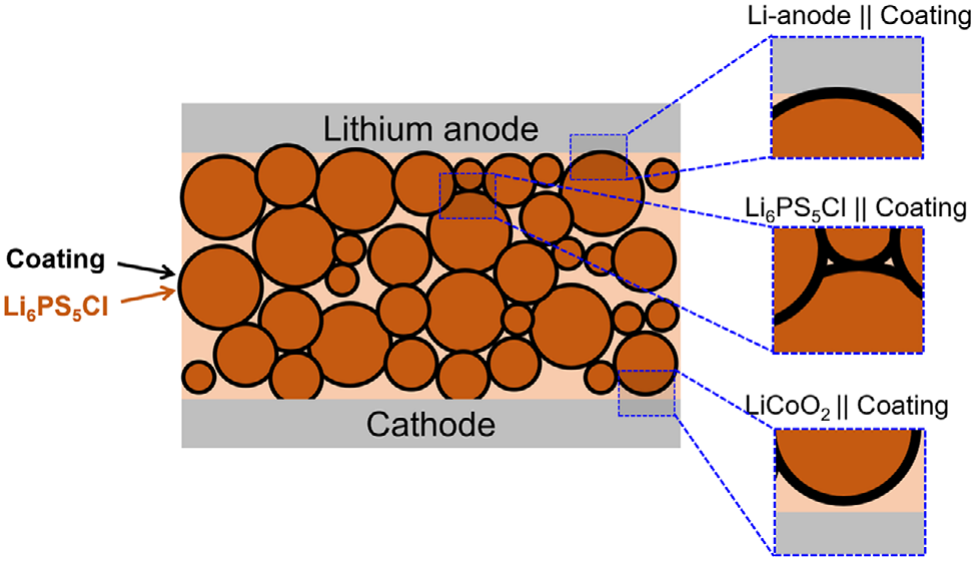
Interfaces modeled in this work. The left image shows the all‐solid‐state battery containing Li_6_PS_5_Cl electrolyte powders (dark orange) coated with ALD‐grown oxides (black). The three smaller schematics on the right are the magnified representations of the Li‐anode || Coating, Li_6_PS_5_Cl || Coating, and Cathode || Coating interfaces.

To identify candidate coatings for the LPSCl SSE, we developed a two‐step process for evaluating their suitability for experimental exploration. In the first step, we evaluate the thermodynamic stability of candidate coatings with three types of interfaces in typical all‐solid‐state batteries: the Electrolyte || Coating interface, the Anode || Coating interface, and the Cathode || Coating interface (**Figure** **1**). As we are focusing on ALD metal oxide coatings in this study, we will refer to the coating as “Oxide” in the following discussion. A broad spectrum of ALD‐synthesizable, binary oxides across the first six rows of the periodic table is considered as coating candidates. Li metal and two representative oxide cathodes (i.e., LiCoO_2_ and LiMn_2_O_4_) are considered as anode and cathode materials, respectively. We also assess the predicted electronic properties of these coating materials by calculating their bandgaps in both bulk and nanoscale geometries to better understand their potential impacts on the electronic conductivity of coated LPSCl.

#### 2.1.1 Electrolyte || Oxide Interface

The reactivity or stability of the LPSCl || Oxide interface was selected as the initial screening criterion.^[^ ^35^ ^]^ The thermodynamic stability of this interface against decomposition reactions was evaluated by calculating the reaction enthalpy, denoted as ΔE, using DFT energies at 0 K for all possible reactions (as described in the Experimental Section). One such reaction between Li_6_PS_5_Cl and Li_2_O is shown in Equation (1), with Δ*E* = −0.278 eV atom^−1^:

(1) $$0.2\;Li_{6}PS_{5}\mathrm{Cl}+0.8\;Li_{2}O\to Li_{2}S+0.2\;Li_{3}PO_{4}+0.2\;\mathrm{LiCl}$$

**Figure 2:**
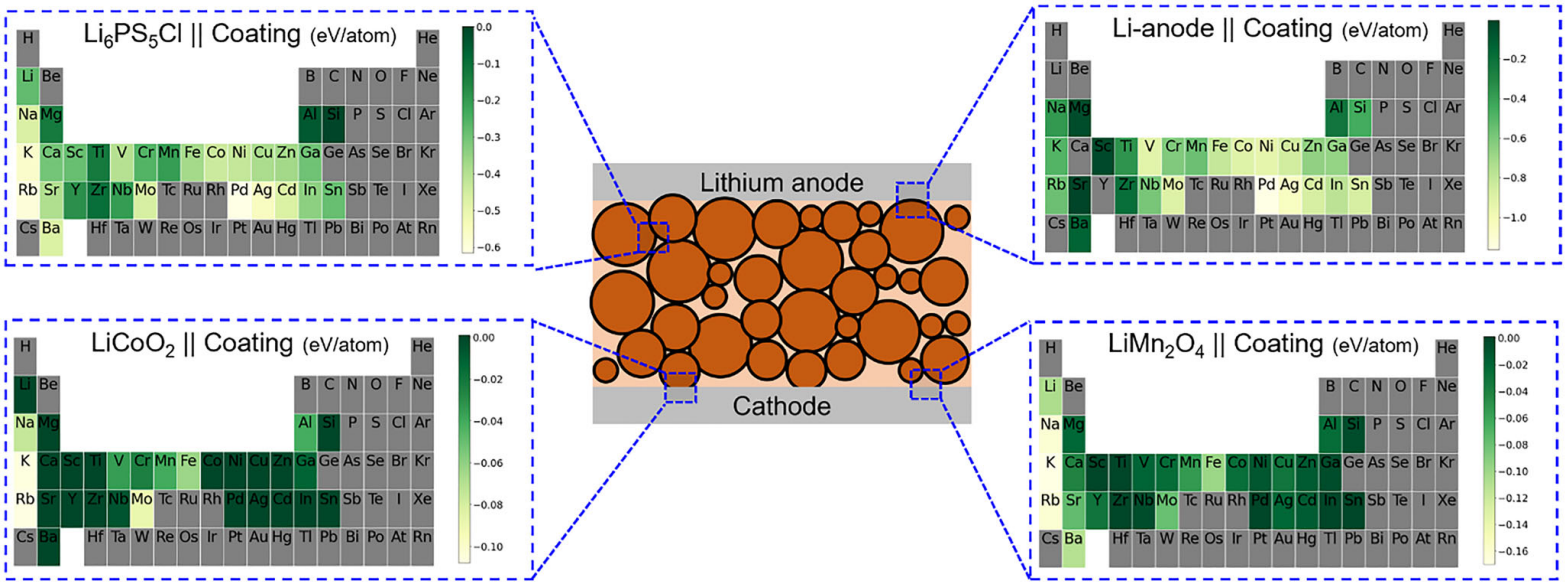
Heatmaps showing the reactivity of various interfaces. The central schematic shows the solid‐state battery shown in Figure 1. In each heatmap, the coating refers to the binary oxide of the corresponding element. In each scale bar, dark green colors indicate less reactive interfaces or more favorable oxides. For oxides that do not react with the cathodes, ΔE values were set to zero. Yellow colors denote elements that form reactive oxide interfaces. Grey denotes elements where the reaction energies were not calculated.

The thermodynamic reaction data for Equation (1) was obtained from the Materials Project database.^[^ ^43^ ^]^ Analogously, for each oxide chemistry, there can be several types of reactions with the LPSCl electrolyte, depending on the stoichiometry (or relative amounts of the oxide and electrolyte). For each oxide type, the electrolyte‐oxide reaction with the largest thermodynamic driving force (most negative value of reaction enthalpy ΔE) was selected to generate the periodic table heatmap in **Figure** **2**. Overall, there are ten oxides forming less reactive LPSCl || Oxide interfaces relative to LPSCl || Li_2_O. The seven most stable oxides identified are SiO_2_, Al_2_O_3_, ZrO_2_, MgO, TiO_2_, Y_2_O_3_, and Nb_2_O_5_, with ΔE values ranging from 0 to −0.2 eV atom^−1^. These correspond to elements with valence electronic configuration s^2^, s^2^p^1^, s^2^p^2^, d^2^, and d^3^ (fully occupied *s*‐orbital, partially occupied *p*‐orbital, and early transition metals). SiO_2_ is predicted to be the most stable with no reaction products at the LPSCl || SiO_2_ interface (Δ*E* = 0). Elements with a single *s*‐orbital valence electron (Li, Na, K, Rb) and high *d*‐orbital occupancies (Fe, Co, Ni, Cu, Zn, Pd, Ag, Cd) form more reactive electrolyte‐oxide interfaces. Of these, Δ*E* (LPSCl || ZnO) = −0.372 eV atom^−1^ is the lowest (least reactive or most stable), and is included as an additional candidate in **Table** **1**. CaO also exhibits a lower ΔE than ZnO (−0.304 eV) and is included as well. We note that elements with *p*‐orbital valence electrons (In, Ga, and Sn) exhibit lower ΔE values than ZnO, but are excluded here due to their unfavorable Li reactivity (described below). A full listing of the LPSCl || Oxide reactions explored is provided in Table S1 (Supporting Information).

**Table 1:** Promising candidates to create stable Li_6_PS_5_Cl || Oxide interfaces. The first ten oxides are those for which the Li_6_PS_5_Cl || Oxide interface is more stable relative to the Li_6_PS_5_Cl || Li_2_O. The reaction between Li_6_PS_5_Cl and Li_2_O is shown in Equation (1), with Δ*E* = −0.278 eV atom^−1^. The last oxide is ZnO, which forms the most stable Li_6_PS_5_Cl || Oxide interface amongst the other elements with *d*‐orbital valence electrons.

Oxide	Li_6_PS_5_Cl || Oxide reaction	ΔE [ eV atom^−1^]
SiO_2_	Li_6_PS_5_Cl + SiO_2_ → No reaction	0.000
Al_2_O_3_	0.2 Li_6_PS_5_Cl + 0.8 Al_2_O_3_ → 0.2 Li_3_PS_4_ + 0.2 LiCl + 0.1 LiAlS_2_ + 0.3 LiAl_5_O_8_	−0.044
ZrO_2_	0.3333 Li_6_PS_5_Cl + 0.6667 ZrO_2_ → 0.3333 Li_2_S + 0.6667 ZrS_2_ + 0.3333 Li_3_PO_4_ + 0.3333 LiCl	−0.097
MgO	0.2 Li_6_PS_5_Cl + 0.8 MgO → 0.2 Li_3_PO_4_ + 0.2 Li_2_S + 0.2 LiCl + 0.8 MgS	−0.125
TiO_2_	0.3333 Li_6_PS_5_Cl + 0.6667 TiO_2_ → 0.1111 Li_4_TiS_4_ + 0.2222 Li(TiS_2_)_2_ + 0.1111 TiS_3_ + 0.3333 LiCl + 0.3333 Li_3_PO_4_	−0.126
Sc_2_O_3_	0.429 Li_6_PS_5_Cl + 0.571 Sc_2_O_3_ → 0.036 Li_3_Sc_2_(PO_4_)_3_ + 1.071 LiScS_2_ + 0.321 Li_3_PO_4_ + 0.429 LiCl	−0.132
Y_2_O_3_	0.4286 Li_6_PS_5_Cl + 0.5714 Y_2_O_3_ → 1.071 LiYS_2_ + 0.07143 YPO_4_ + 0.3571 Li_3_PO_4_ + 0.4286 LiCl	−0.176
Nb_2_O_5_	0.5556 Li_6_PS_5_Cl + 0.4444 Nb_2_O_5_ → 0.5614 NbS_3_ + 0.5556 Li_3_PO_4_ + 0.04678 Li_5_(NbS_2_)_7_ + 0.4386 Li_2_S + 0.5556 LiCl	−0.200
MnO	0.2 Li_6_PS_5_Cl + 0.8 MnO → 0.06667 Li_6_MnS_4_ + 0.7333 MnS + 0.2 Li_3_PO_4_ + 0.2 LiCl	−0.202
Cr_2_O_3_	0.4286 Li_6_PS_5_Cl + 0.5714 Cr_2_O_3_ → 0.8571 LiCrS_2_ + 0.4286 Li_3_PO_4_ + 0.1429 Cr_2_S_3_ + 0.4286 LiCl	−0.256
CaO	0.2 Li_6_PS_5_Cl + 0.8 CaO → 0.2 Li_2_S + 0.2 Li_3_PO_4_ + 0.8 CaS + 0.2 LiCl	−0.304
ZnO	0.2 Li_6_PS_5_Cl + 0.8 ZnO → 0.2 Li_2_S + 0.2 Li_3_PO_4_ + 0.8 ZnS + 0.2 LiCl	−0.372

#### 2.1.2 Anode || Oxide Interface

It is equally important to consider the reactivity or stability of the Li || Oxide interface. As with the LPSCl || Oxide interface, we screened different oxides as potential coatings to mitigate chemical reactions between Li metal and the SSE. For example, Equation (2) shows the reaction between Li and Al_2_O_3_, which was the focus of our previous study.

(2) $$0.6667\;\mathrm{Li}+0.3333\;Al_{2}O_{3}\to 0.1667\;\mathrm{LiAl}+0.5\;\mathrm{LiAl}O_{2}$$

Similar to the previous set of interface reaction calculations, for each oxide type, the anode‐oxide reaction with the largest thermodynamic driving force (most negative value of reaction enthalpy ΔE) was selected to generate the periodic table heatmap shown in Figure 2. **Table** **2** lists the Li || Oxide reactions with ΔE values for all candidate oxides identified in Table 1, and a full listing of the reactions explored is provided in Table S2 (Supporting Information). Among these, ZnO is the most reactive with Li, with a ΔE value of −0.653 eV atom^−1^, and it is set as the cutoff point for consideration. As with the LPSCl || Oxide interfaces, the driving forces for the reaction between Li and other *d*‐block oxides (metals with partial *d*‐orbital occupancies) are much stronger than with ZnO, shown by the yellow color scale in Figure 2. There are five additional oxides (Na_2_O, K_2_O, Rb_2_O, SrO, and BaO) not appearing in Table 1 for which the reaction with Li is less favorable than for ZnO. However, these oxides all react more strongly with LPSCl than ZnO (Table S1, Supporting Information), and they are therefore excluded from consideration here. Combining the data in Tables 1 and 2, MgO, ZrO_2_, and Al_2_O_3_ are predicted to be relatively stable against both LPSCl and Li metal. In our previous work, ALD coating of LPSCl electrolyte powders with Al_2_O_3_ was found to improve the chemical stability of LPSCl in contact with metallic Li.^[^ ^13^ ^]^ Tables 1 and 2 reinforce these results by establishing Al_2_O_3_ as a promising oxide coating with small thermodynamic driving forces for reactions.

**Table 2:** Li || Oxide interfaces with the lowest driving forces for interface reactions. Li_2_O was not chosen as the reference oxide since there is no reaction with Li. Instead, all oxides from Table 1 and any other oxides with intermediate reaction energy values are listed in Table 2. The reference is ZnO, which is the least stable in Table 1.

Oxide	Li‐anode || Oxide reaction	ΔE [eV atom^−1^]
Y_2_O_3_	Li + Y_2_O_3_ → No reaction	0.000
CaO	Li + CaO → No reaction	0.000
Sc_2_O_3_	0.6 Li + 0.4 Sc_2_O_3_ → 0.6 LiScO_2_ + 0.2 Sc	−0.034
MgO	0.75 Li + 0.25 MgO → 0.25 Li_2_O + 0.25 LiMg	−0.040
ZrO_2_	0.6122 Li + 0.3878 ZrO_2_ → 0.06122 Zr_3_O + 0.102 Li_6_Zr_2_O_7_	−0.185
Al_2_O_3_	0.6667 Li + 0.3333 Al_2_O_3_ → 0.1667 LiAl + 0.5 LiAlO_2_	−0.220
TiO_2_	0.6316 Li + 0.3684 TiO_2_ → 0.1053 Ti_2_O + 0.1579 Li_4_TiO_4_	−0.357
SiO_2_	0.6667 Li + 0.3333 SiO_2_ → 0.1667 Li_4_SiO_4_ + 0.1667 Si	−0.447
MnO	0.6667 Li + 0.3333 MnO → 0.3333 Li_2_O + 0.3333 Mn	−0.557
Nb_2_O_5_	0.8 Li + 0.2 Nb_2_O_5_ → 0.4 LiNbO_2_ + 0.2 Li_2_O	−0.585
Cr_2_O_3_	0.8571 Li + 0.1429 Cr_2_O_3_ → 0.4286 Li_2_O + 0.2857 Cr	−0.611
ZnO	0.7 Li + 0.3 ZnO → 0.1 LiZn_3_ + 0.3 Li_2_O	−0.653

#### 2.1.3 Cathode || Oxide Interface

To interrogate the stability of the Cathode || Oxide interface, two common cathode materials, LiCoO_2_ (LCO) and LiMn_2_O_4_ (LMO), are modeled in this work. ΔE values for oxides that do not react with the cathodes are set to zero. We note that for both LMO and LCO, the calculated ΔE ranges are significantly smaller than those calculated for the LPSCl || Oxide and Li || Oxide interfaces. As an example, the most favorable alumina reaction with LMO:

(3) $$\begin{array}{ccc} 0.1525\;\mathrm{LiM}n_{2}O_{4} & + & 0.8475\;Al_{2}O_{3}\to 0.1017\;Mn_{3}O_{4}\\ & & +0.08475\;Al_{11}O_{18}+0.1525\;\mathrm{LiA}l_{5}O_{8} \end{array}$$

has reaction energy of −0.024 eV atom^−1^ – an order of magnitude smaller than the reaction with Li metal – and is unlikely to proceed because of kinetic limitations. In general, most of the oxides considered are predicted to be stable against LMO and LCO (Tables S3 and S4, Supporting Information), suggesting that coated LPSCl should exhibit enhanced stability against oxide cathode materials. However, given the relatively small difference in reactivity predicted between all coatings, we do not anticipate interfacial reactivity with the cathode to be a reliable predictor of performance, at least for oxide‐based coatings.

Finally, we note that all reactions considered in this analysis are two‐phase reactions, as these likely contribute most significantly to the stability of the interface. However, it is possible that three‐phase reactions (e.g., Li‐Oxide‐LPSCl) also contribute, and a discussion of a representative three‐phase reaction for Li‐Al_2_O_3_‐LPSCl is included in the Supporting Information. In this specific case, no significant differences from the two‐phase analysis are observed; however, this is an important area to consider in the future.

### 2.2 Electronic Properties of Candidate Coating Chemistries

**Figure 3:**
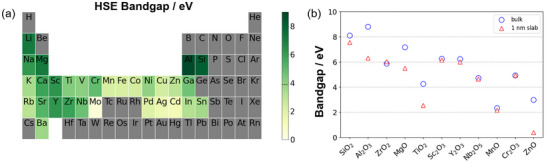
a) Heatmap showing the bandgap of the corresponding bulk binary oxide computed at the HSE level of DFT (mixing parameter = 0.32). The dashed black line at 3.92 eV denotes the bandgap of bulk LPSCl. b) Bulk and thin film bandgaps of the candidate oxides listed in Table 1, computed at the HSE level of DFT.

Having determined the thermodynamic stability of possible oxide coatings at various interfaces, it is also important to assess the electronic properties of these candidate materials, as coatings that are too electronically conductive can significantly degrade the properties of SSE separators. The bandgap of pure LPSCl was calculated to be 3.92 eV using the HSE06 exchange‐correlation functional. To evaluate overall trends in electronic properties, the heatmap in **Figure** **3**a shows the bandgaps of the corresponding bulk oxides, also calculated at the HSE level of theory. The corresponding values used to generate the heatmap are summarized in Table S5 (Supporting Information). Al_2_O_3,_ SiO_2_, and MgO have the largest calculated bandgaps of 8.80, 8.12, and 7.16 eV, respectively. Consistent with this large bandgap, Al_2_O_3_‐coated LPSCl was demonstrated to have reduced electronic conductivity in our previous work.^[^ ^13^ ^]^ Notably, all of the oxides listed in Table 1, except MnO, have larger bandgaps than LPSCl. There are various other candidates with larger bandgaps as well; however, they generally form highly reactive interfaces and are not considered further for experimental study here.

Whereas the heatmap in Figure 3a shows the trends in bandgaps of various bulk oxides, nanoscale coatings may exhibit different electronic properties from the bulk calculations. In particular, the electronic conductivity of metal oxide thin film coatings can be strongly influenced by defects such as metal interstitials and oxygen vacancies. For instance, ZnO has an experimental bandgap of 3.40 eV – similar to LPSCl – but the electronic conductivity of ALD ZnO coatings is typically high due to oxygen vacancy defects.^[^ ^44^ ^]^ Therefore, additional calculations were done for the oxides listed in Table 1, using ≈1 nm slabs to mimic the ALD coating thicknesses considered here (Figure 3b). Nanoscale SiO_2_, Al_2_O_3_, ZrO_2_, Sc_2_O_3_, and Y_2_O_3_ films retain large bandgaps over 6 eV, and MgO and Cr_2_O_3_ are also insulating with ≈5 eV bandgaps. The bandgaps of TiO_2_ and MnO films are ≈2 eV, which is less than the bulk gap of LPSCl. Whereas bulk ZnO has a bandgap of ≈3 eV, the bandgap reduces to 0.4 eV for a 1 nm film. We note that bandgaps of 1 nm films have not been previously reported; however, the bulk bandgaps calculated here (using a mixing parameter of 0.32) generally agree well with the literature.^[^ ^45^, ^46^, ^47^ ^]^ Additionally, reported bandgaps of monolayer oxides also suggest that they can be effective in suppressing electron conduction.^[^ ^48^ ^]^ Interestingly a higher bandgap of 3.5 eV was previously predicted for monolayer ZnO,^[^ ^49^ ^]^ and this discrepancy is likely due to the known sensitivity of this particular oxide to its specific defect chemistry, mentioned above. Taken together, these calculations suggest that coating the LPSCl electrolyte with oxides having smaller bandgaps relative to LPSCl, such as TiO_2_, MnO, and ZnO, may increase the overall electronic conductivity, whereas large bandgap oxides can suppress electron transport. Among these, SiO_2_, Al_2_O_3_, ZrO_2_, and MgO are promising for experimental investigation since they also form the least reactive interfaces.

### 2.3 Ionic and Electronic Conduction through Interfacial Decomposition Products

**Figure 4:**
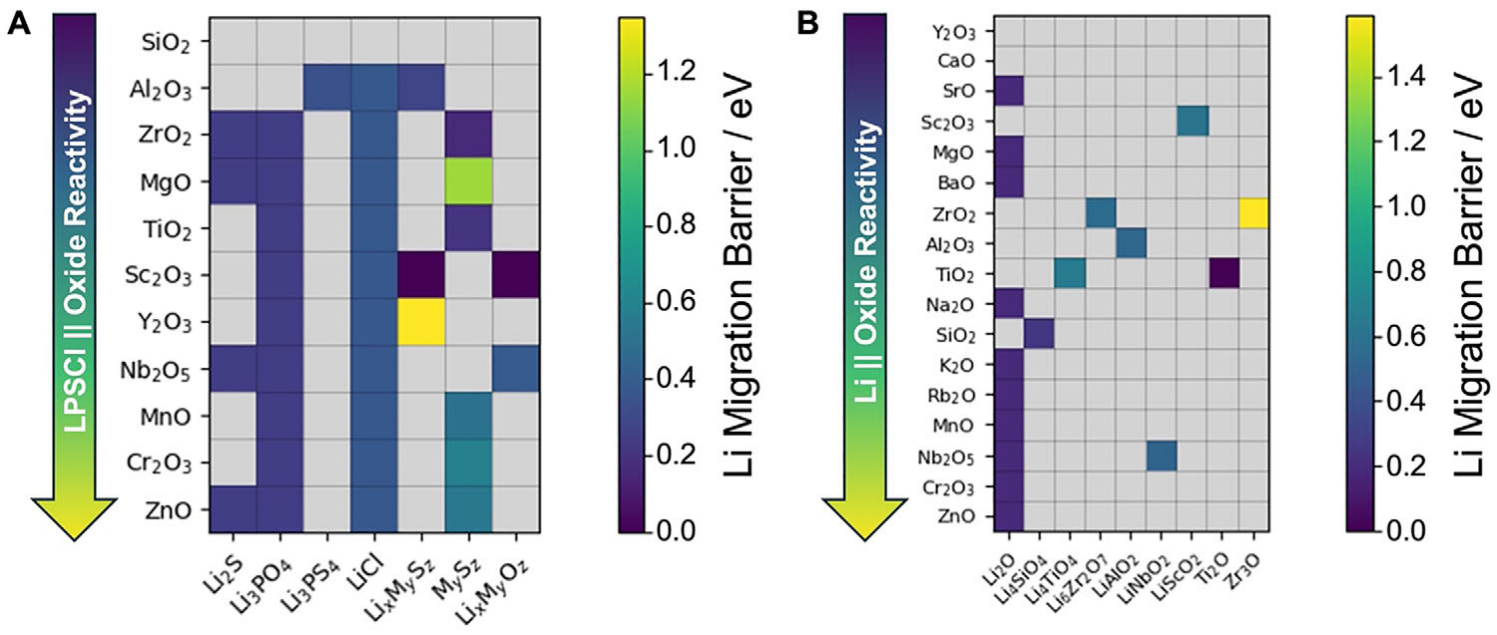
Heatmaps showing the lithium migration barrier values in various reaction products formed at the a) LPSCl || Oxide interface and b) Li || Oxide interface. The downward arrows to the left of the heatmaps denote the direction of increasing interfacial reactivity for each metal oxide.

In addition to the coatings themselves, the ionic and electronic conductivity of decomposition products that form at the various interfaces considered can also influence Li transport. As a result, the second step of our screening methodology analyzes the bandgap and migration barrier for Li transport through several key reaction products predicted by the DFT analysis performed in the first step. It should be noted here that reaction products formed at the interface between bulk LPSCl particles and thin film oxides are likely not to retain their bulk structure. However, comparing the Li‐ion migration barriers through these compounds is a useful heuristic for screening. **Figure** **4** summarizes the various types of reaction products considered, colored according to the Li‐ion migration barrier through the bulk form of those reaction products. The exact values calculated are summarized in Tables S6 and S7 (Supporting Information). The arrow on the left of each graph indicates the direction of increasing interfacial reactivity for each oxide.

For all reactions at the LPSCl || Oxide interface (Figure 4a), LPSCl primarily decomposes as Li_3_PO_4_, Li_3_PS_4_, Li_2_S, and LiCl. These compounds are Li^+^ conductors –, e.g., Li_2_S and LiCl allow relatively fast lithium migration along the <100> and <110> directions.^[^ ^13^ ^]^ Migration barriers in Li_3_PO_4_ (0.25 eV), Li_3_PS_4_ (0.34–0.41 eV), and LiAl_5_O_8_ (0.28–0.33 eV) were obtained from previously reported ab initio molecular dynamics simulations.^[^ ^50^, ^51^, ^52^, ^53^ ^]^ Many of the other reaction products are layered hexagonal sulfides that exhibit a wide range of Li^+^ migration barriers (e.g., Li(TiS_2_)_2_, ZrS_2_, Cr_2_S_3_, LiCrS_2_, LiScS_2_, Li_5_(NbS_2_)_7_, LiYS_2_, TiS_3_, NbS_3_). These products are denoted as Li_x_M_y_S_z_ and M_y_S_z_ in Figure 4a. In all cases, the displacement path is parallel to the (0001) plane. Lithium migration in other compounds can occur in three dimensions (e.g., Li_2_S and LiCl, noted above), with Li_4_TiS_4_ and Li_6_MnS_4_ also exhibiting low migration barriers of 0.30 and 0.24 eV, respectively. In contrast, the close‐packed structures of MgS and YPO_4_ are strongly resistant to lithium conduction, with barriers over 1 eV. Additionally, Figure 4b presents the Li migration barriers through the non‐metallic reaction products at the Li || Oxide interface, listed in Table 2. Li_2_O is the most common product and supports fast Li conduction. Notably, Zr_3_O exhibits a large barrier of 1.4 eV for lithium migration, potentially indicative of poor transport characteristics in ZrO_2_‐coated LPSCl.

Along with their Li^+^ transport properties, comparing the bandgap of oxide and sulfide reaction products can also indicate the impact of the coating on electronic conductivity. Tables S5 and S6 (Supporting Information) also summarize the bandgap of the oxide and sulfide compounds formed in the reactions listed in Table 1. Encouragingly, the main reaction products calculated at the LPSCl || Oxide interface (Li_3_PO_4_, Li_3_PS_4_, Li_2_S, and LiCl) also have bandgaps larger than that of bulk LPSCl, suggesting that oxide coatings should generally reduce electronic conductivity. LiAlS_2_, LiAl_5_O_8_, and MgS have particularly large bandgaps relative to LPSCl, further supporting Al_2_O_3_ and MgO as promising candidates. Ga_2_O_3_, In_2_O_3_, and ZnO, which are predicted to be moderately reactive at both the LPSCl || Oxide and Li || Oxide interfaces, result in metal sulfides and decomposition products with bulk bandgaps comparable to that of LPSCl. The sulfides MnS, NbS_3_, TiS_3_, and Li(TiS_2_)_2_ are almost metallic, and Zr_3_O is metallic, which suggests potentially increased electronic conductivities upon coating with MnO, Nb_2_O_5_, TiO_2,_ and ZrO_2_.

Although the trends described above are not quantitative, the heuristics outlined by our computational screening approach clearly suggest some candidates for experimental exploration. Encouragingly, Al_2_O_3_ is identified as a promising coating candidate, in line with our previous experimental demonstration of its multifaceted benefits to stability and performance. MgO is also identified as a promising candidate due to its similarly favorable reaction profile and ionic/electronic properties relative to Al_2_O_3_. Overall, Al_2_O_3_ and MgO coatings are predicted to i) reduce chemical degradation due to low driving forces for their decomposition reactions, ii) provide lithium conduction channels through the decomposition products, and iii) lower electronic conductivity due to the relatively large bandgaps of the oxides and their various reaction products. In order to better understand the interplay between oxide coating stability and the ionic/electronic conductivity of their reaction products, ZrO_2_ and ZnO were also selected for further experimental investigation. In the case of ZrO_2_, its predicted reactivity with LPSCl lies in between that of Al_2_O_3_ and MgO; however, it may not yield the same electrochemical performance due to unfavorable Li^+^ transport through its reaction products that also generally exhibit low bandgaps, which may increase electronic conductivity. ZnO was selected due to its higher predicted reactivity and electronic conductivity relative to Al_2_O_3_, MgO, and ZrO_2_, and its otherwise favorable Li^+^ transport and reduced electronic conductivity for predicted reaction products.

### 2.4 ALD Coating of LPSCl by Computationally Identified Materials of Interest

**Figure 5:**
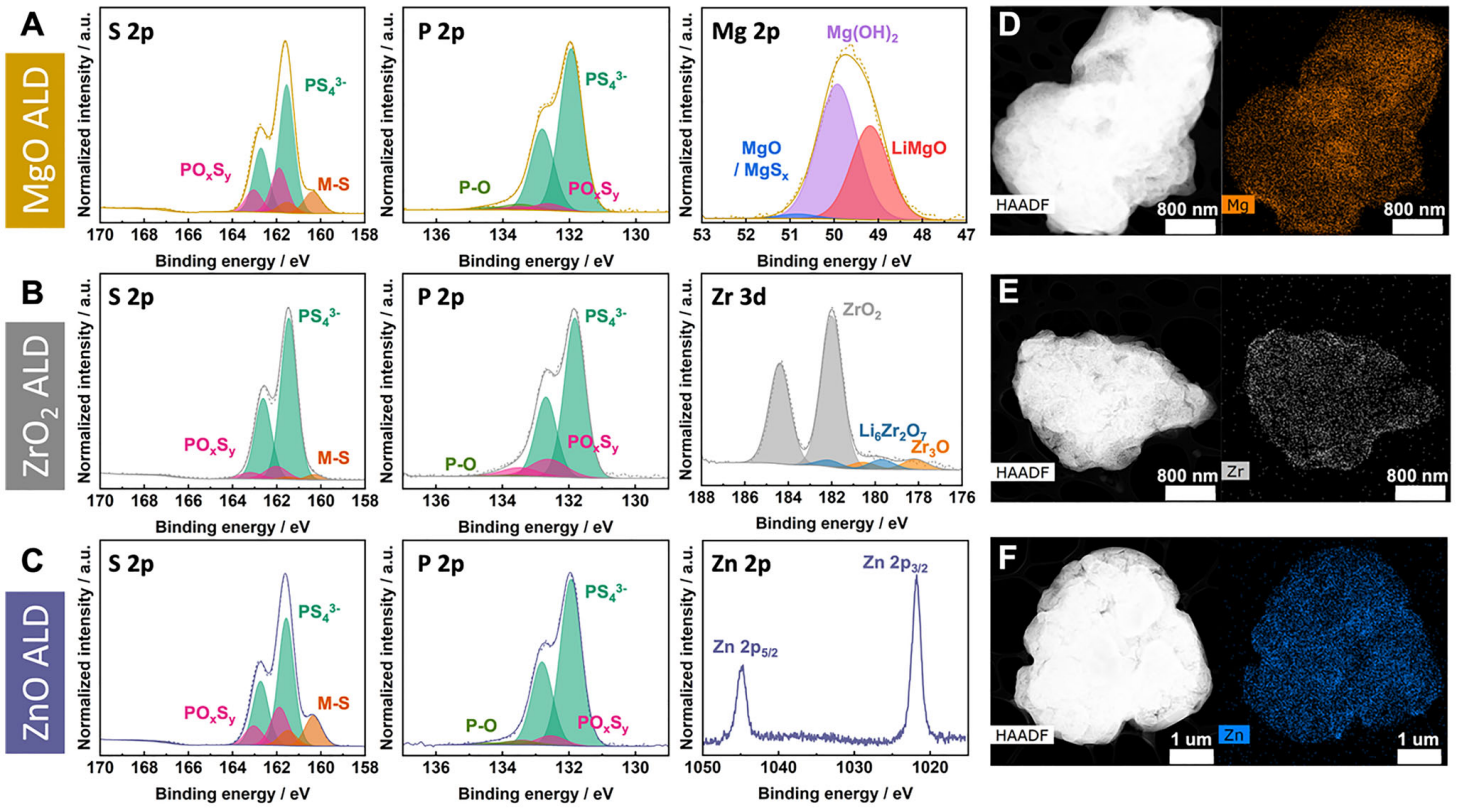
Physicochemical properties of ALD‐coated LPSCl powders. XPS core level spectra of S 2p, P 2p, and corresponding coating elements A) Mg 2p, B) Zr 3d, and C) Zn 2p. Measurements were performed on pellets pressed from coated powders. The thickness of the ALD layer on individual powders is estimated to be ≈1 nm. STEM images and EDS elemental mapping of D) Mg, E) Zr, and F) Zn signal on coated LPSCl powders.

Having identified multiple coating chemistries of interest, we turn our attention to experimental validation of the computational predictions. XPS measurements were performed to interrogate the resulting surface chemistry of ALD‐coated LPSCl (**Figure** **5**a–c). These XPS data were collected on dense pellets prepared from ALD‐coated powders (see Experimental Section for details). Comparing the surface chemistry of coated materials with the pristine LPSCl (Figure S1, Supporting Information) confirms successful deposition of coating layers with negligible reactivity of the underlying LPSCl in all cases. X‐ray diffraction measurements (Figure S2, Supporting Information) also indicate no changes to the underlying bulk material after coating. Detailed XPS peak analysis parameters for each coating chemistry are tabulated in Table S8 (Supporting Information), and each specific chemistry will be discussed in detail below. Scanning transmission electron microscopy (STEM) and energy dispersive spectroscopy (EDS) further confirm the conformality of the different ALD coating chemistries on LPSCl powders (Figure 5d–f; Figure S3, Supporting Information), which is maintained for both thick and thin coatings (Figure S4, Supporting Information). An even distribution of the metal oxide over the LPSCl particle is observed for all coated materials, suggesting there is no preferential nucleation or segregation of the ALD coating. Notably, as expected from a thin, conformal coating, a slight increase in the Mg, Zr, and to a lesser extent Zn concentrations is observed near the particle surface, however, the sensitivity of LPSCl to the electron beam limits more in depth interfacial STEM analysis.

In Figure 5a, the MgO‐coated LPSCl exhibits predominant features at 161.6 and 162.7 eV in the S 2p and 132.0 and 132.8 eV in the P 2p core level spectra that correspond to PS_4_^3−^ units in the underlying LPSCl, consistent with minimal reactivity of the underlying material after ALD coating. We also observe a minor feature corresponding to oxysulfide species (PO_x_S_y_) in both the S 2p (161.9 and 163.0 eV) and P 2p (132.6 and 133.5 eV) core level spectra, in addition to a weaker signal of P─O bonding at 133.5 and 134.4 eV in the P 2p core level, which probably results from minor oxidation of LPSCl during handling or upon exposure to H_2_O during ALD.^[^ ^54^, ^55^ ^]^ Analysis of the Mg 2p core level reveals a more reduced Mg bonding environment (49.9 eV) relative to what would be expected for Mg‐O or Mg‐S (50.8 eV), which are observed as minority species.^[^ ^56^, ^57^ ^]^ The binding energy (BE) of this primary Mg feature is more consistent with Mg(OH)_2_^[^ ^56^ ^]^ as seen previously for ALD MgO deposited using H_2_O.^[^ ^58^ ^]^ It is also possible this species is related to a more covalent Mg oxysulfide and/or weakly lithiated oxide/sulfide phase relative to purely ionic MgO/MgS, which would result in a peak to lower binding energy as well. Analysis of the O 1s core level (Figure S5, Supporting Information) indicates the presence of a metal oxide feature that would not be expected from Mg(OH)_2_, suggesting a mixture of oxide and hydroxide species in the ALD coating. Interestingly, we also observe a feature at even lower BE (49.2 eV) in the Mg 2p core level, which we ascribe to LiMgO, suggesting the interfacial reaction products are a mixture of those predicted by DFT for the LPSCl || MgO and the Li || MgO interfaces.

Similar to MgO, ZrO_2_‐coated LPSCl exhibits minimal reactivity in the S 2p and P 2p core levels (Figure 5b). The Zr 3d core level reveals a doublet at 182.0 and 184.4 eV corresponding to ZrO_2_ as the dominant phase, along with features to lower BE that are consistent with small amounts of Li_6_Zr_2_O_7_ (179.7 and 182.1 eV) and Zr_3_O (178.2 and 180.6 eV).^[^ ^20^ ^]^ This suggests that the ZrO_2_ ALD layer also grows on LPSCl without significant reactivity with the underlying LPSCl, and that it forms reaction products that are more consistent with the DFT predictions summarized above for the Li || ZrO_2_ interface. Interestingly, no observable coating is obtained for ALD ZnO when attempting to deposit directly on LPSCl (Figure S6, Supporting Information). It is possible that this is due to the higher reactivity predicted by the calculations discussed above, leading to volatile decomposition products and/or the inability of ZnO to bind effectively to the LPSCl surface. It may also simply be due to unfavorable surface chemistry between LPSCl and the diethyl zinc precursor used in the ALD process. In fact, the low reactivity of diethyl zinc toward non‐hydroxylated surfaces is key to the success of selective area ZnO ALD.^[^ ^59^ ^]^ However, deposition of a single layer of ALD Al_2_O_3_ (≈1 Å) is sufficient to induce growth of ZnO films, resulting in a clear Zn signal in the XPS analysis (Figure 5c). We also observe a slight increase in the metal sulfide species in the S 2p core level on the ZnO‐coated LPSCl as compared to the pristine material (Figure S1, Supporting Information). Detailed analysis indicates this is consistent with a small amount of ZnS formation (Figure S7, Supporting Information), in line with thermodynamic stability predictions discussed above. Overall, each of the coating chemistries explored exhibits minimal reactivity with the underlying LPSCl, with the observed reaction products in line with those predicted at both the LPSCl || Oxide and Li || Oxide interfaces. This indicates that either the reactions between the oxide coatings and LPSCl are kinetically limited, or that the nature of the ALD process itself kinetically stabilizes the interface, perhaps due to the specific chemistries of the ALD precursors utilized. Furthermore, this indicates that thermodynamic reactivity may not be a sufficiently reliable indicator of the resulting (electro)chemical properties of coated materials.

**Figure 6:**
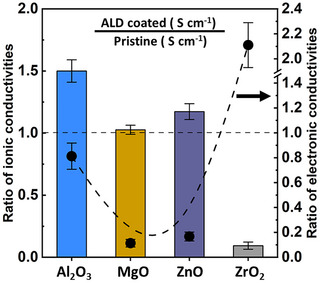
Ionic and Electronic conductivity of ALD‐coated LPSCl relative to uncoated LPSCl. The dotted line intersects the y‐axis at a value of 1. As noted in the text, the ALD ZnO‐coated LPSCl has a 1Å Al_2_O_3_ seed layer. All values measured at 25 °C, 5 MPa fixed stack pressure.

To investigate the impact of the ALD coating chemistry on ionic and electronic conductivity, we utilized a combination of electrochemical impedance spectroscopy (EIS) and chronoamperometry (CA). Details concerning the cell construction and analysis can be found in the Experimental Section. Figure S8 (Supporting Information) compares the measured ionic conductivity of LPSCl before and after coating with ≈1 nm each of the four ALD chemistries (Al_2_O_3_, MgO, ZnO, and ZrO_2_), and Figures S9 and S10 (Supporting Information) display DC polarization and temperature‐dependent EIS data used to generate these plots. Although there are differences in the ionic conductivity of the starting material due to batch‐to‐batch variability in the LPSCl synthesis, these results clearly demonstrate the influence of ALD chemistry on Li^+^ and electronic conductivity. In **Figure** **6**, we summarize the relative change in ionic and electronic conductivity, which normalizes for batch‐to‐batch variations and enables a direct comparison of the effects of each of the four coating chemistries on the resulting transport properties of pellets pressed from coated powders. ALD Al_2_O_3_‐coated LPSCl exhibits the largest increase in ionic conductivity (a ratio of ≈1.5 for coated vs uncoated), consistent with our previous results.^[^ ^13^ ^]^ ALD ZnO coatings also yield improved ionic conductivity, albeit more moderately (ratio of ≈1.2), while ALD MgO coatings exhibit little to no change. The increase in ionic conductivity for ZnO‐based coatings is likely driven by similar effects as reported in our previous work,^[^ ^13^ ^]^ where ab‐initio molecular dynamics simulations suggested enhanced vacancy‐mediated Li^+^ transport at grain boundaries could take place due to the formation of Li‐S‐Li‐O‐Li‐S‐Li networks in Al_2_O_3_‐based coatings. We note that in that work, 1 Å Al_2_O_3_ coatings (identical to the seed layer used to induce ZnO growth) yielded no changes to ionic conductivity, suggesting this enhancement is due to the ZnO coating itself. In the case of ALD ZrO_2_ coatings, we observe an order of magnitude reduction in the ionic conductivity (from 1.26 to 0.12 mS cm^−1^), consistent with the DFT predictions of low Li^+^ mobility through ZrO_2_‐based reaction products such as Li_6_Zr_2_O_7_ and Zr_3_O, both of which are observed by XPS.

MgO and ZnO coatings yield significant decreases in electronic conductivity as well – up to an order of magnitude relative to uncoated LPSCl (ratios of 0.11 and 0.17, respectively). We again note that the 1 Å seed layer of Al_2_O_3_ necessary to nucleate the ZnO coating cannot explain this dramatic decrease in electronic conductivity, as minimal differences in electronic conductivity for 1Å Al_2_O_3_ coatings were observed previously, indicating this is a property of the ZnO coating itself. This result is surprising given that ALD ZnO is predicted to have the highest electronic conductivity of the coatings examined in this study and is often utilized as a transparent conducting layer.^[^ ^44^, ^60^ ^]^ The pronounced drop in electronic conductivity of the LPSCl following ZnO ALD likely relates to the much lower electronic conductivity of the interfacial reaction products. For instance, ALD ZnS has a resistivity ≈10^8^ times higher compared to ALD ZnO.^[^ ^61^ ^]^ A more modest reduction in electronic conductivity was measured for ALD Al_2_O_3_ (0.81), consistent with our previous results for 1 nm coatings and further supporting the conclusion that the electronic properties of ZnO‐coated LPSCl derive from the ZnO and not the much thinner Al_2_O_3_ seed layer. In contrast, ALD ZrO_2_ coatings result in a factor of two increase in electronic conductivity, consistent with the predictions above that ZrO_2_‐based reaction products should exhibit bandgaps lower than that of LPSCl, yielding higher electronic conductivity.

Overall, these conductivity results suggest that the ionic and electronic properties of the DFT‐calculated reaction products are significantly more predictive metrics of performance than the thermodynamic stability of a given oxide coating. This is despite the relatively low fraction of reaction products observed via XPS analysis, suggesting that even small amounts of interfacial reaction product formation can dramatically alter the resulting electrochemical properties of the coated LPSCl. Given its favorable Li^+^ conductivity, significantly reduced electronic conductivity, and more straightforward ALD processing relative to ZnO, we selected MgO‐coated LPSCl for more detailed investigations of (electro)chemical stability and performance.

### 2.5 (Electro)Chemical Stability of MgO‐Coated LPSCl

**Figure 7:**
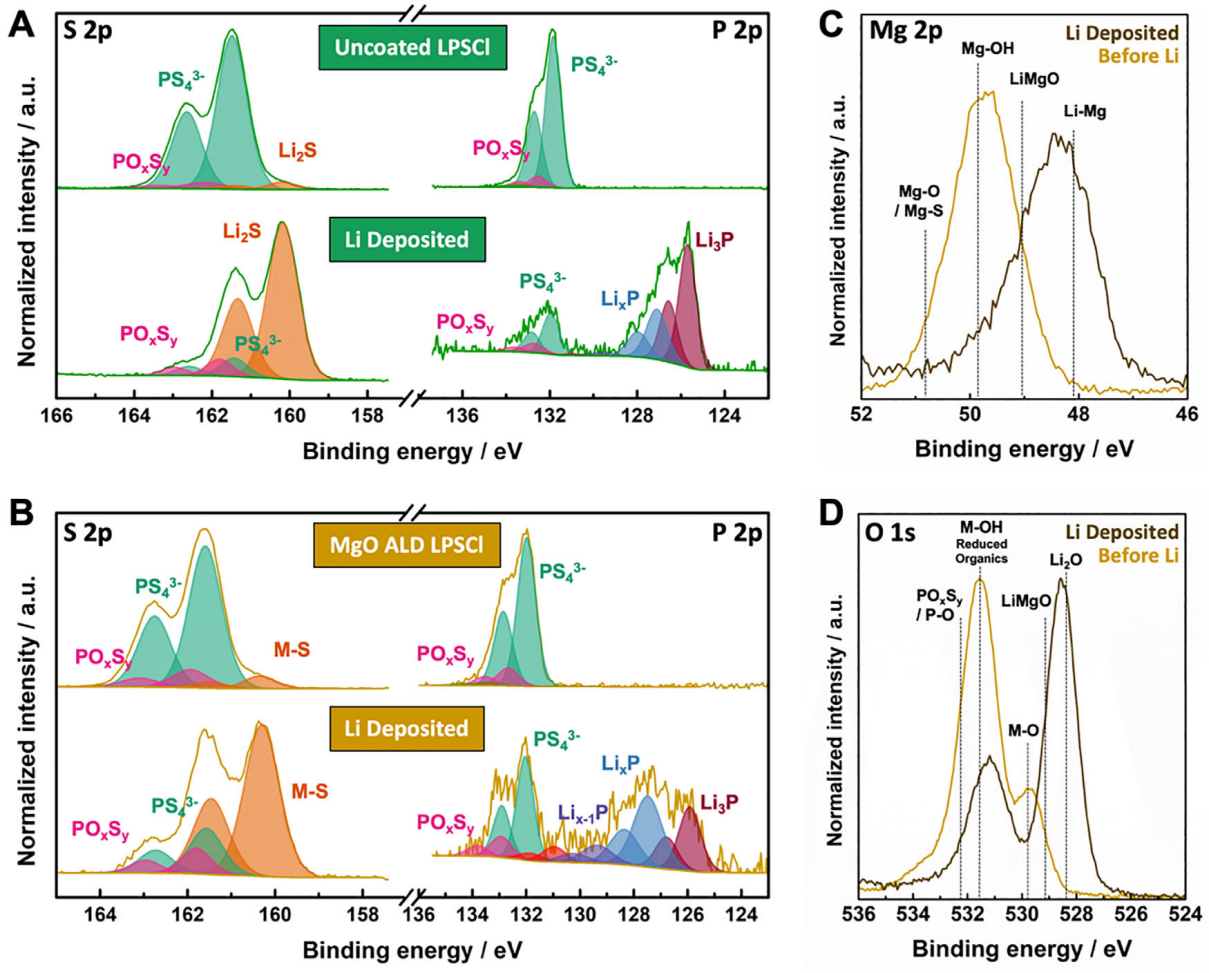
Chemical and electrochemical reactivity of the MgO ALD LPSCl against Li contact. A,B) S 2p and P 2p core level spectra of uncoated and ALD MgO‐coated LPSCl before and after Li deposition. C) Mg 2p and D) O 1s core level spectra of ALD MgO‐coated LPSCl before and after Li deposition.

Similar to our previous results on Al_2_O_3_‐coated materials and consistent with the DFT predictions above, the chemical compatibility of LPSCl against Li metal is substantially improved with ALD MgO coating (**Figure** **7**). XPS analysis was performed on both pristine and ALD MgO‐coated LPSCl pellets before and after Li deposition to understand differences in chemical reactivity (see Experimental Section for details). Core level fitting parameters and quantitative analysis of relative peak ratios are provided in Table S9 (Supporting Information). Consistent with previous reports,^[^ ^13^, ^62^ ^]^ significant reactivity of uncoated LPSCl with metallic Li is observed (Figure 7a, as indicated by reduction of PS_4_^3−^ tetrahedral units in the S 2p core level (161.5 and 162.6 eV) to Li_2_S (160.3 and 161.4 eV), and the formation of Li_3_P (125.7 and 126.6 eV) and other reduced phosphorous species in the P 2p core level. In contrast, the ALD MgO‐coated LPSCl exhibits a lower extent of reduction to Li_2_S in the S 2p core level, indicating enhanced stability against Li metal. This is even more apparent in the P 2p core level spectra, with a much lower fraction of Li_3_P relative to PS_4_^3−^ and a higher fraction of less reduced Li_3‐x_P species. These results are similar to our previous observations with Al_2_O_3_‐coated materials, clearly demonstrating the power of our computationally guided approach to predict candidate coating materials with improved chemical stability against Li metal.

In addition to the significantly reduced Li reactivity of MgO‐coated LPSCl, we also find that the reaction chemistry of the ALD MgO layer is consistent with the DFT predictions described above. Specifically, analysis of the Mg 2p core level spectra before and after Li deposition (Figure 7c) reveals that the entire Mg 2p peak envelope shifts to lower BE. Deconvolution indicates a transition from primarily Mg oxide/hydroxide species to a mixture of Li‐Mg alloy and LiMgO – where Li may substitute Mg cations in the MgO matrix.^[^ ^63^, ^64^, ^65^ ^]^ Analysis of the O 1s core level (Figure 7d) also reveals a shift in the MgO metal oxide feature to lower BE that is consistent with our assignment of LiMgO in the Mg 2p core level. It further reveals the formation of Li_2_O, which is also predicted to form by DFT. It is important to highlight that small amounts of LiMgO are also observed on the as‐prepared MgO ALD LPSCl (Figure 5a), and the persistence of LiMgO even after exposure to Li metal suggests that reaction of the MgO layer may be kinetically limited, consistent with other observations above. Overall, the extent of lithiation of the MgO layer is greater than that observed for Al_2_O_3_‐coated materials, suggesting the possibility of improved interfacial properties of MgO‐coated materials in contact with Li metal.

To compare the electrochemical stability of MgO and uncoated LPSCl at different current densities, we prepared symmetric Li‐Li cells of each material (see Experimental Section for details of cell assembly). We utilized a galvanostatic critical current density (CCD) protocol where 0.25 mAh cm^−2^ of lithium was stripped and plated at current densities of 0.1–0.9 mA cm^−2^, increasing in steps of 0.05 mA cm^−2^. The resistance of the cell was measured using EIS during every other cycle. A schematic of the protocol is shown in Figure S11 (Supporting Information), and similar protocols have been previously reported to support differences in electrochemical stability in LPSCl.^[^ ^66^, ^67^, ^68^ ^]^ In addition, each cell was cycled once at 0.3 mA cm^−2^ (0.25 mAh cm^−2^ per half cycle) prior to the CCD measurement to homogenize the Li distribution at each interface and yield more reproducible cell response (see Figures S12 and S13 and Supporting Information for details).

**Figure 8:**
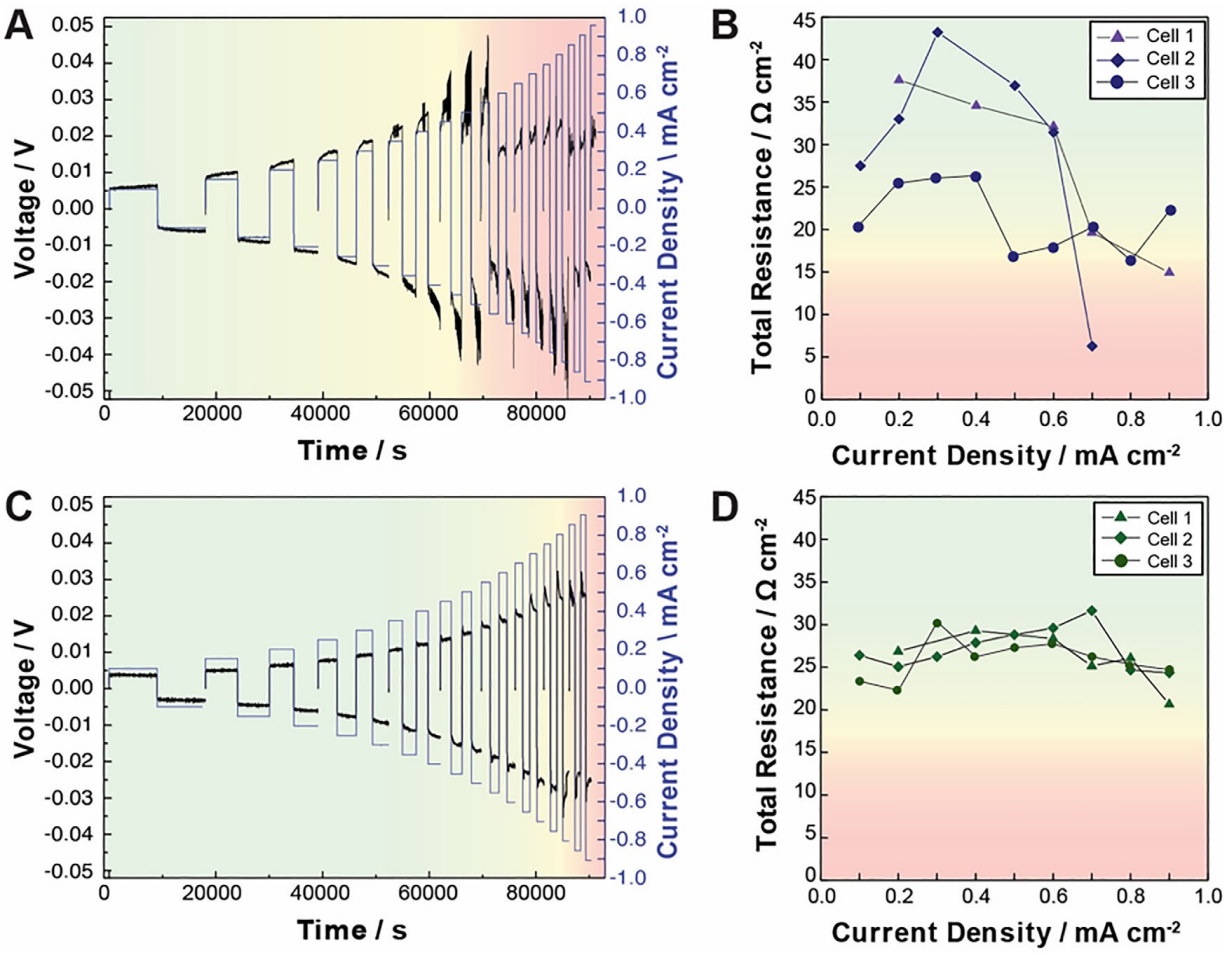
CCD measurements of Li || Li symmetric cells with uncoated and MgO‐coated LPSCl. A) Voltage response of uncoated LPSCl from 0.1 to 0.9 mA cm^−2^ and B) corresponding ASR measured after the indicated current density step. C) Voltage response of ALD MgO‐coated LPSCl from 0.1 to 0.9 mA cm^−2^ and D) corresponding ASR measured after the indicated current density step. All measurements performed at 25 °C, 5 MPa fixed stack pressure using 20 µm Li foils. CCD measurements in A and C correspond to Cell 1 in panels B and D.

Utilizing this protocol, several notable differences between the electrochemical response of coated and uncoated LPSCl are observed. First, uncoated LPSCl exhibits a clear decoupling of the potential response at 0.5 mA cm^−2^ (**Figure** **8**a; Figures S14a and S15a, Supporting Information), in line with previous CCD measurements of uncoated LPSCl.^[^ ^13^, ^66^, ^69^, ^70^ ^]^ This voltage decoupling is correlated with a precipitous drop in the area‐specific resistance (ASR) at ≈0.6 mA cm^−2^ (Figure 8b) that is consistent with a hard short of the cell. In contrast, the MgO‐coated material shows a largely stable voltage response up to 0.9 mA cm^−2^ (Figure 8c; Figures S14b and S15b, Supporting Information), with some slight voltage decoupling initiating at ≈0.8 mA cm^−2^. Interestingly, no significant changes in ASR are observed up to 0.9 mA cm^−2^ (Figure 8d), suggesting that the initial stages of voltage decoupling observed for MgO‐coated materials are likely due to soft, rather than hard, shorts. We note that similar improvements in CCD were observed for Al_2_O_3_‐coated powders in our previous work, indicating MgO coatings provide similar benefits to the electrochemical stability of coated LPSCl. Furthermore, although full cell cycling is beyond the scope of this report, we have previously demonstrated benefits to performance from Al_2_O_3_‐coated LPSCl,^[^ ^71^ ^]^ and we fully anticipate similar benefits from the coatings reported in this work. Finally, the overpotentials measured in the MgO‐coated LPSCl are lower than the uncoated material (consistent with the lower ASR values measured in these materials), which suggests that the LiMgO and LiMg alloy formation predicted by computational modeling and observed in XPS does indeed improve the interfacial properties of MgO‐coated LPSCl with Li metal. Taken together, these measurements demonstrate the power of our combined computational and experimental approach to design new coating chemistries for LPSCl powders, with MgO identified and demonstrated to significantly improve the (electro)chemical stability of coated materials.

## 3 Conclusion

In this work, we developed a combined computational and experimental approach to discovering and demonstrating ALD‐based oxide coatings of LPSCl powders that result in significantly improved (electro)chemical properties relative to uncoated materials. We utilized a systematic two‐step computational screening approach to evaluate reaction energies of various solid‐solid interfaces, bandgaps of bulk and thin‐film oxides, and ionic and electronic conductivity of reaction products, to identify three new candidate coatings – MgO, ZrO_2_, and ZnO – with a range of predicted stability, ionic/electronic conductivity, and reaction product properties. Encouragingly, this screening methodology also highlighted Al_2_O_3_ as a promising coating, which we had previously demonstrated experimentally to provide multifaceted benefits to SSE performance. All three predicted coating chemistries were successfully deposited via ALD as conformal layers on LPSCl powders. Coated materials were shown to exhibit minimal chemical reactivity with the underlying LPSCl, and yielded reaction products consistent with those predicted by DFT. MgO and ZnO‐based coatings further exhibited enhanced ionic and reduced electronic conductivity, whereas ZrO_2_‐based coatings were shown to reduce the ionic and increase the electronic conductivity of coated LPSCl. Based on these results and those of the DFT screening methodology, we propose that the most predictive metric for coating performance is the ionic and electronic conductivity of the calculated LPSCl || Oxide and Li || Oxide reaction products, with materials that favor the formation of conductive oxides and alloys with Li metal in particular yielding the most favorable properties. The intrinsic stability of the oxide coatings themselves was found to be less predictive of performance, as the most reactive coating investigated (ZnO) also yielded multiple favorable properties. Detailed investigations of MgO‐coated LPSCl revealed improved stability against Li metal, as well as significant improvements to both ASR and CCD, highlighting this material as a particularly promising candidate for further exploration and scale‐up. In general, these results highlight the vast chemical space available for exploring new powder coating chemistries for sulfide‐based SSEs, and they provide clear design rules for developing coated materials with enhanced stability, processability, and performance for next‐generation solid‐state batteries. It is plausible that many additional beneficial coating chemistries exist beyond simple oxides, and we anticipate a new research frontier for exploring the myriad benefits of SSE powder coating to improve the stability, processability, and performance of sulfide‐based solid‐state batteries.

## 4 Experimental Section

### Reaction Energy Calculations

Data were retrieved from the Materials Project database using the Selenium WebDriver. Specifically, the Interface Reactions app of the legacy Materials Project was used to collect the data for Figure 2. For each combination of reactants (oxide + LPSCl, oxide + Li, oxide + cathode), all possible reactions were calculated, and the reaction with the largest driving force (highest negative value of the reaction enthalpy) was used to create the periodic table maps.

### DFT Calculations

Bandgap calculations were done using the Vienna Ab initio Simulation Package (VASP),^[^ ^72^ ^]^ employing the exchange‐correlation functional of Heyd, Scuseria, and Ernzerhof (HSE06),^[^ ^73^ ^]^ with a mixing parameter of 0.32. The kinetic energy cutoff for the plane wave basis set was set to 500 eV. The convergence criteria for total energy and ionic forces were set to 10^−6^ eV and 10^−2^ eV A^−1^, respectively. Bandgap calculations of ≈1 nm oxide slabs were done after introducing a ≈1 nm vacuum region in the supercells. Migration energy barriers were calculated using the climbing image nudged elastic band (CI‐NEB) formalism,^[^ ^74^ ^]^ by generating intermediate replicas using linear interpolation of initial and final states. The exchange‐correlation functional of Perdew–Burke–Ernzerhof (PBE) was used for these calculations.^[^ ^75^ ^]^

### LPSCl Powder Synthesis

Argyrodite solid‐state electrolytes with the nominal composition of Li_6_PS_5_Cl were synthesized via a conventional solid‐state reaction route, a widely adopted method known for producing phase‐pure, high‐crystallinity materials suitable for solid‐state battery applications. Stoichiometric or near‐stoichiometric amounts of high‐purity precursors (lithium chloride (LiCl), lithium sulfide (Li_2_S), and phosphorus pentasulfide (P_2_S_5_), all sourced from Sigma–Aldrich) were weighed and thoroughly mixed under an inert argon atmosphere to prevent any reaction with moisture or oxygen, which can degrade sulfide‐based precursors. The resulting powder mixture was pressed into compact pellets using a uniaxial press at an applied pressure of ≈14 MPa to promote good interparticle contact and efficient solid‐state diffusion. These pellets were then sealed in an evacuated or argon‐filled vessel and thermally treated at 550 °C for 3 h to initiate and complete the solid‐state reaction. This annealing process enabled the formation of the crystalline argyrodite phase. After cooling, the reacted pellets were manually ground and subjected to high‐energy ball milling in a planetary mill to reduce the particle size to below 10 µm. The phase purity and crystallinity of the synthesized Li_6_PS_5_Cl were confirmed by XRD analysis (Figure S2, Supporting Information). The diffraction patterns matched well with the reference patterns for the cubic argyrodite phase, with no evidence of secondary phases or unreacted precursors, indicating successful synthesis. The final powders were handled and stored in an argon‐filled glovebox to prevent degradation due to the material's sensitivity to air and moisture.

### ALD on the LPSCl Powders

ALD coating was performed using a custom hot‐walled, viscous flow tubular ALD reactor integrated to a glove box described previously.^[^ ^76^, ^77^ ^]^ The LPSCl powder samples (≈1 g) were contained in a shallow, stainless steel tray with a wire cloth cover to provide easy access to the ALD precursor vapors.^[^ ^78^ ^]^ The powder sample was loaded into the ALD reactor along with Si (100) witness coupons for measuring the film thickness after deposition using spectroscopic ellipsometry. For XPS Li reactivity studies, a pre‐pressed LPSCl pellet was loaded in the reactor along with the powders to generate a model surface under the same deposition conditions used to generate powders for electrochemical testing. The samples were allowed to equilibrate to the growth conditions for ≈15 mins prior to growth. Typical growth conditions were 150 °C and 1.5 Torr pressure of ultrahigh purity Ar supplied from a liquid Ar tank via mass flow controllers at 90 sccm. The ALD precursors used were trimethylaluminum (TMA, Strem, 98%), bis(cyclopentadienyl) magnesium (MgCp_2_, Strem, 99.9%), tetrakis(dimethylamino) zirconium(IV) (TDMAZr, Strem, 98%), and diethyl zinc (DEZ, Strem, 95%). The deposition conditions are listed in Table S10 (Supporting Information). As described in the main text, the ZnO ALD was preceded by 1 cycle Al_2_O_3_ ALD to nucleate the ZnO ALD. Without this nucleation treatment, no signal for Zn was observed by XPS on the LPSCl powder.

### X‐Ray Photoelectron Spectroscopy (XPS)

Pellets from the pristine and the coated LPSCl powders were used for XPS analysis. A total of 40 mg of the powders were pressed into pellets using a hydraulic press with 250 MPa at room temperature in an Ar‐filled glove box using a customized 6 mm pellet press die set. Polypropylene membranes were used to avoid surface contamination from the stainless‐steel plunger during pressing. The prepared samples were all mounted in a glove box and transferred through an interconnected ultra‐high vacuum linear transfer system (10^−10^ mbar) to either the characterization or physical vapor deposition (PVD) module, which eliminates possible exposure to air. To measure the chemical reactivity of the samples against Li^0^, a thin layer of Li metal was applied on the surface of the pellets via electron beam evaporation in the PVD module, and the coated samples were subsequently transferred to the characterization module for subsequent XPS measurements. XPS was carried out using a Specs PHOIBOS 150 hemispherical energy analyzer with a monochromatic Al Ka X‐ray source (1486.7 eV) at 10^−9^ mbar of vacuum level. The survey spectra were measured using a pass energy of 40 eV at a resolution of 0.2 eV per step and a total integration time of 0.1 s per point. The core level spectra were collected using a pass energy of 20 eV at a resolution of 0.05 eV per step and a total integration time of 0.5 s per point. Peak analysis was performed using CasaXPS software with a Shirley‐type background and 70–30 Gaussian–Lorentzian peak shapes. Energy calibration was performed using Cl 2p_3/2_ core level peak at 198.8 eV. The samples were grounded to the spectrometer via conductive carbon tape or Cu clips, and an electron flood gun was used to compensate for surface charging.

### STEM‐EDS

Scanning transmission electron microscopy (STEM) and X‐ray energy dispersive spectroscopy (EDS) were performed on an FEI Talos F200X equipped with an EDS detector (Bruker). Elemental maps were collected with a STEM spot size of 6. All S/TEM specimens were prepared by directly depositing powders onto carbon‐coated copper grids. To reduce electron beam related artifacts, the coated particles were characterized at dose rates <10 e As^−2^.

### Blocking and Li Symmetric Cell Assembly

Symmetric cells of Li||SSE||Li were assembled by using a pressed cell and cycled at different current densities (0.05–0.9 mA cm^−2^) inside of a glovebox at room temperature. LPSCl pellets were prepared by applying 675 MPa to 100 mg of material for 120 s. Li‐coated Cu foil was then pressed onto the LPSCl for 600s with a pressure of 5 MPa. Ionic conductivity measurements were performed at variable temperatures (−20–80 °C) for Arrhenius activation energy measurements. CCD measurements were performed at 25 °C. In all cases 5 MPa stack pressure was applied, which was fixed prior to electrochemical testing.

### Electrochemical Impedance Spectroscopy Measurements

Electrochemical impedance spectroscopy (EIS) measurements were conducted using a Biologic potentiostat, with a frequency range spanning from 7 MHz to 1 Hz and an applied amplitude of 100 mV. Blocking cells of Al/C||SSE||C/Al were prepared by pressing 100 mg of material and blocking electrodes, single sided carbon coated Al, at a pressure of 675 MPa for 120 s. Cells were prepared inside a glovebox at room temperature. The EIS measurements were conducted in a temperature controlled environmental chamber, within air‐tight, pressurized cells.

## Conflict of Interest

J.G.C., J.W.E., Z.D.H., A.U.M., P.Z., and A.S. have filed a patent application related to the ALD coating and computational screening approaches detailed in this work.

## Supporting information



Supporting Information

## Data Availability

The data that support the findings of this study are available from the corresponding author upon reasonable request.
