# Supplementary material for: Computationally‐Guided Development of Sulfide Solid Electrolyte Powder Coatings for Enhanced Stability and Performance of Solid‐State Batteries
Source: Adv Sci (Weinh). 2025 Oct 15;12(47):e13191. doi: 10.1002/advs.202513191 (PMC12713078; doi:10.1002/advs.202513191)
Supplement: Supplementary file 1 — Supporting Information [file ADVS-12-e13191-s001.docx]

# Computationally-Guided Development of Sulfide Solid Electrolyte Powder Coatings for Enhanced Stability and Performance of Solid-State Batteries

*Aditya Sundar^1,†^, Taewoo Kim^1,†^, Francisco Lagunas^2^, Anil U. Mane^2^, Udochukwu D. Eze^2^, Colton M. Ginter,^2,3^* *Rajesh Pathak^2^, Sanja Tepavcevic^1^, Jeffrey W. Elam^2^, Zachary D. Hood^2^, Peter Zapol^1,‡^, Justin G. Connell^1,*^*

*^1^ Materials Science Division, Argonne National Laboratory, 9700 S Cass Ave., Lemont, IL 60439*

*^2^ Applied Materials Division, Argonne National Laboratory, 9700 S Cass Ave., Lemont, IL 60439*

*^3^ Pritzker School of Molecular Engineering, The University of Chicago, Chicago, IL 60637*

*^†^Indicates equal contribution*

*^‡^email: zapol@anl.gov*

**email: jconnell@anl.gov*

**Table S1.** DFT-calculated reactions and thermodynamic reaction energies at LPSCl || Oxide interfaces for all oxides considered in Figure 2.

| **Oxide** | **LPSCl \|\| Oxide Reaction** | $\boldsymbol{\Delta}$**E (eV/atom)** |
| --- | --- | --- |
| Ag_2_O | 0.1111 Li_6_PS_5_Cl + 0.8889 Ag_2_O → 0.1111 Li_3_PO_4_ + 0.4444 Ag_2_S + 0.1111 LiCl + 0.1111 Li_2_SO_4_ + 0.8889 Ag | -0.549 |
| Al_2_O_3_ | 0.2 Li_6_PS_5_Cl + 0.8 Al_2_O_3_ → 0.2 Li_3_PS_4_ + 0.2 LiCl + 0.1 LiAlS_2_ + 0.3 LiAl_5_O_8_ | -0.044 |
| BaO | 0.2 Li_6_PS_5_Cl + 0.8 BaO → 0.1 BaCl_2_ + 0.3 Li_2_S + 0.7 BaS + 0.2 Li_3_PO_4_ | -0.466 |
| CaO | 0.2 Li_6_PS_5_Cl + 0.8 CaO → 0.2 Li_2_S + 0.2 Li_3_PO_4_ + 0.8 CaS + 0.2 LiCl | -0.304 |
| CdO | 0.2 Li_6_PS_5_Cl + 0.8 CdO → 0.2 Li_2_S + 0.2 Li_3_PO_4_ + 0.8 CdS + 0.2 LiCl | -0.466 |
| CoO | 0.1818 Li_6_PS_5_Cl + 0.8182 CoO → 0.1591 Li_2_S + 0.1818 Li_3_PO_4_ +  0.09091 Co_9_S_8_ + 0.1818 LiCl + 0.02273 Li_2_SO_4_ | -0.438 |
| Cr_2_O_3_ | 0.4286 Li_6_PS_5_Cl + 0.5714 Cr_2_O_3_ → 0.8571 LiCrS2 + 0.4286 Li3PO4 +  0.1429 Cr2S3 + 0.4286 LiCl | -0.256 |
| Cu_2_O | 0.2 Li_6_PS_5_Cl + 0.8 Cu_2_O → 0.1333 Li_3_CuS_2_ + 0.1833 Cu_7_S_4_ + 0.2 Li_3_PO_4_ +  0.2 LiCl + 0.1833 Cu | -0.384 |
| FeO | 0.2 Li_6_PS_5_Cl + 0.8 FeO → 0.2 Li_2_S + 0.2 Li_3_PO_4_ + 0.8 FeS + 0.2 LiCl | -0.374 |
| Ga_2_O_3_ | 0.4286 Li_6_PS_5_Cl + 0.5714 Ga_2_O_3_ → 0.1429 Ga_2_S_3_ + 0.4286 Li_3_PO_4_ +  0.8571 LiGaS_2_ + 0.4286 LiCl | -0.296 |
| In_2_O_3_ | 0.4286 Li_6_PS_5_Cl + 0.5714 In_2_O_3_ → 0.4286 Li_3_PO_4_ + 0.8571 LiInS_2_ + 0.4286 LiCl + 0.1429 In_2_S_3_ | -0.340 |
| K_2_O | 0.2 Li_6_PS_5_Cl + 0.8 K_2_O → 0.4 K_2_S + 0.2 Li_3_PO_4_ + 0.6 KLiS + 0.2 KCl | -0.569 |
| Li_2_O | 0.2 Li_6_PS_5_Cl + 0.8 Li_2_O → Li_2_S + 0.2 Li_3_PO_4_ + 0.2 LiCl | -0.278 |
| MgO | 0.2 Li_6_PS_5_Cl + 0.8 MgO → 0.2 Li_3_PO_4_ + 0.2 Li_2_S + 0.2 LiCl + 0.8 MgS | -0.125 |
| MnO | 0.2 Li_6_PS_5_Cl + 0.8 MnO → 0.06667 Li_6_MnS_4_ + 0.7333 MnS + 0.2 Li_3_PO_4_ +  0.2 LiCl | -0.202 |
| MoO_2_ | 0.3333 Li_6_PS_5_Cl + 0.6667 MoO_2_ → 0.3333 Li_3_PO_4_ + 0.6667 MoS_2_ + 0.3333 Li_2_S + 0.3333 LiCl | -0.459 |
| Na_2_O | 0.2 Li_6_PS_5_Cl + 0.8 Na_2_O → 0.6 NaLiS + 0.2 Li_3_PO_4_ + 0.2 NaCl + 0.4 Na_2_S | -0.462 |
| Nb_2_O_5_ | 0.5556 Li_6_PS_5_Cl + 0.4444 Nb_2_O_5_ → 0.5614 NbS_3_ + 0.5556 Li_3_PO_4_ +  0.04678 Li_5_(NbS_2_)_7_ + 0.4386 Li_2_S + 0.5556 LiCl | -0.200 |
| NiO | 0.1818 Li_6_PS_5_Cl + 0.8182 NiO → 0.1591 Li_2_S + 0.09091 Ni_9_S_8_ + 0.1818 Li_3_PO_4_ + 0.1818 LiCl + 0.02273 Li_2_SO_4_ | -0.424 |
| PdO | 0.1111 Li_6_PS_5_Cl + 0.8889 PdO → 0.04938 Pd_16_S_7_ + 0.09877 PdS +  0.1111 Li_3_PO_4_ + 0.1111 LiCl + 0.1111 Li_2_SO_4_ | -0.615 |
| Rb_2_O | 0.2 Li_6_PS_5_Cl + 0.8 Rb_2_O → 0.2 Li_3_PO_4_ + 0.4 Rb_2_S + 0.6 RbLiS + 0.2 RbCl | -0.593 |
| Sc_2_O_3_ | 0.3333 Li_6_PS_5_Cl + 0.6667 SnO_2_ → 0.5 SnS_2_ + 0.1667 Li_4_SnS_4_ + 0.3333 LiCl +  0.3333 Li_3_PO_4_ | -0.285 |
| SnO_2_ | 0.3333 Li_6_PS_5_Cl + 0.6667 SnO_2_ → 0.5 SnS_2_ + 0.1667 Li_4_SnS_4_ + 0.3333 LiCl +  0.3333 Li_3_PO_4_ | -0.285 |
| SrO | 0.2 Li_6_PS_5_Cl + 0.8 SrO → 0.8 SrS + 0.2 LiCl + 0.2 Li_2_S + 0.2 Li_3_PO_4_ | -0.390 |
| TiO_2_ | 0.3333 Li_6_PS_5_Cl + 0.6667 TiO_2_ → 0.1111 Li_4_TiS_4_ + 0.2222 Li(TiS_2_)_2_ + 0.1111 TiS_3_ + 0.3333 LiCl + 0.3333 Li_3_PO_4_ | -0.126 |
| V_2_O_5_ | 0.4146 Li_6_PS_5_Cl + 0.5854 V_2_O_5_ → 0.9756 VS_2_ + 0.1951 Li_3_VO_4_ + 0.122 Li_2_SO_4_ + 0.4146 LiCl + 0.4146 Li_3_PO_4_ | -0.401 |
| Y_2_O_3_ | 0.4286 Li_6_PS_5_Cl + 0.5714 Y_2_O_3_ → 1.071 LiYS_2_ + 0.07143 YPO_4_ + 0.3571 Li_3_PO_4_ + 0.4286 LiCl | -0.176 |
| ZnO | 0.2 Li_6_PS_5_Cl + 0.8 ZnO → 0.2 Li_2_S + 0.2 Li_3_PO_4_ + 0.8 ZnS + 0.2 LiCl | -0.372 |
| ZrO_2_ | 0.3333 Li_6_PS_5_Cl + 0.6667 ZrO_2_ → 0.3333 Li_2_S + 0.6667 ZrS_2_ + 0.3333 Li_3_PO_4_ +  0.3333 LiCl | -0.097 |
| SiO_2_ | Li_6_PS_5_Cl + SiO_2_ → No reaction | 0.000 |

**Table S2.** DFT-calculated reactions and thermodynamic reaction energies at Li || Oxide interfaces for all oxides considered in Figure 2.

| **Oxide** | **Li \|\| Oxide Reaction** | $\boldsymbol{\Delta}$**E (eV/atom)** |
| --- | --- | --- |
| Ag_2_O | 0.6667 Li + 0.3333 Ag_2_O → 0.3333 Li_2_O + 0.6667 Ag | -1.043 |
| Al_2_O_3_ | 0.6667 Li + 0.3333 Al_2_O_3_ → 0.1667 LiAl + 0.5 LiAlO_2_ | -0.220 |
| BaO | 0.6667 Li + 0.3333 BaO → 0.3333 Li_2_O + 0.3333 Ba | -0.134 |
| CaO | 0.5 Li + CaO → No Reaction | 0.000 |
| CdO | 0.6667 Li + 0.3333 CdO → 0.3333 Li_2_O + 0.3333 Cd | -0.864 |
| CoO | 0.6667 Li + 0.3333 CoO → 0.3333 Li_2_O + 0.3333 Co | -0.904 |
| Cr_2_O_3_ | 0.8571 Li + 0.1429 Cr_2_O_3_ → 0.4286 Li_2_O + 0.2857 Cr | -0.611 |
| Cu_2_O | 0.6667 Li + 0.3333 Cu_2_O → 0.3333 Li_2_O + 0.6667 Cu | -0.850 |
| FeO | 0.6667 Li + 0.3333 FeO → 0.3333 Li_2_O + 0.3333 Fe | -0.805 |
| Ga_2_O_3_ | 0.8679 Li + 0.1321 Ga_2_O_3_ → 0.3962 Li_2_O + 0.03774 Li_2_Ga_7_ | -0.665 |
| In_2_O_3_ | 0.8571 Li + 0.1429 In_2_O_3_ → 0.4286 Li_2_O + 0.2857 In | -0.779 |
| K_2_O | 0.6667 Li + 0.3333 K_2_O → 0.3333 Li_2_O + 0.6667 K | -0.488 |
| Li_2_O | 0.5 Li + Li_2_O → No Reaction | 0.000 |
| MgO | 0.75 Li + 0.25 MgO → 0.25 Li_2_O + 0.25 LiMg | -0.040 |
| MnO | 0.6667 Li + 0.3333 MnO → 0.3333 Li_2_O + 0.3333 Mn | -0.557 |
| MoO_2_ | 0.8 Li + 0.2 MoO_2_ → 0.4 Li_2_O + 0.2 Mo | -0.901 |
| Na_2_O | 0.6667 Li + 0.3333 Na_2_O → 0.3333 Li_2_O + 0.6667 Na | -0.374 |
| Nb_2_O_5_ | 0.8 Li + 0.2 Nb_2_O_5_ → 0.2 Li_2_O + 0.4 LiNbO_2_ | -0.585 |
| NiO | 0.6667 Li + 0.3333 NiO → 0.3333 Li_2_O + 0.3333 Ni | -0.937 |
| PdO | 0.6818 Li + 0.3182 PdO → 0.3182 Li_2_O + 0.04545 LiPd_7_ | -1.162 |
| Rb_2_O | 0.6667 Li + 0.3333 Rb_2_O → 0.3333 Li_2_O + 0.6667 Rb | -0.557 |
| Sc_2_O_3_ | 0.6 Li + 0.4 Sc_2_O_3_ → 0.6 LiScO_2_ + 0.2 Sc | -0.034 |
| SnO_2_ | 0.8 Li + 0.2 SnO_2_ → 0.4 Li_2_O + 0.2 Sn | -0.864 |
| SrO | 0.6667 Li + 0.3333 SrO → 0.3333 Li_2_O + 0.3333 Sr | -0.008 |
| TiO_2_ | 0.6316 Li + 0.3684 TiO_2_ → 0.1053 Ti_2_O + 0.1579 Li_4_TiO_4_ | -0.357 |
| V_2_O_5_ | 0.8 Li + 0.2 V_2_O_5_ → 0.2 Li_2_O + 0.4 LiVO_2_ | -0.919 |
| Y_2_O_3_ | 0.5 Li + Y_2_O_3_ → No Reaction | 0.000 |
| ZnO | 0.7 Li + 0.3 ZnO → 0.3 Li_2_O + 0.1 LiZn_3_ | -0.653 |
| ZrO_2_ | 0.6122 Li + 0.3878 ZrO_2_ → 0.06122 Zr_3_O + 0.102 Li_6_Zr_2_O_7_ | -0.185 |
| SiO_2_ | 0.6667 Li + 0.3333 SiO_2_ → 0.1667 Li_4_SiO_4_ + 0.1667 Si | -0.447 |

**Table S3.** DFT-calculated reactions and thermodynamic reaction energies at LiCoO_2_ || Oxide interfaces for all oxides considered in Figure 2.

| **Oxide** | **LiCoO_2_ \|\| Oxide Reaction** | $\boldsymbol{\Delta}$**E (eV/atom)** |
| --- | --- | --- |
| Ag_2_O | LiCoO_2_ + AgO → No Reaction | 0.000 |
| Al_2_O_3_ | 0.2308 LiCoO_2_ + 0.7692 Al_2_O_3_ → 0.07692 Al_11_O_18_ + 0.2308 Al_2_CoO_4_ + 0.2308 LiAlO_2_ | -0.041 |
| BaO | LiCoO_2_ + BaO → No Reaction | 0.000 |
| CaO | LiCoO_2_ + CaO → No Reaction | 0.000 |
| CdO | LiCoO_2_ + CdO → No Reaction | 0.000 |
| CoO | LiCoO_2_ + CoO → No Reaction | 0.000 |
| Cr_2_O_3_ | 0.5714 Cr_2_O_3_ + 0.4286 LiCoO_2_ → 0.4286 Cr_2_CoO_4_ + 0.1429 LiCrO_2_ + 0.1429 Li_2_CrO_4_ | -0.024 |
| Cu_2_O | LiCoO_2_ + Cu_2_O → No Reaction | 0.000 |
| FeO | 0.5 FeO + 0.5 LiCoO_2_ → 0.5 LiFeO_2_ + 0.5 CoO | -0.063 |
| Ga_2_O_3_ | 0.6667 Ga_2_O_3_ + 0.3333 LiCoO_2_ → 0.2222 LiGa_5_O_8_ + 0.1111 Ga_2_CoO_4_ + 0.1111 Li(CoO_2_)_2_ | -0.011 |
| In_2_O_3_ | LiCoO_2_ + In_2_O_3_ → No Reaction | 0.000 |
| K_2_O | 0.5714 K_2_O + 0.4286 LiCoO_2_ → 0.1429 K_2_Li_3_CoO_4_ + 0.07143 K_6_Co_2_O_7_ + 0.07143 K_6_Co_2_O_5_ | -0.106 |
| Li_2_O | LiCoO_2_ + Li_2_O → No Reaction | 0.000 |
| MgO | LiCoO_2_ + MgO → No Reaction | 0.000 |
| MnO | 0.5 MnO + 0.5 LiCoO_2_ → 0.5 LiMnO_2_ + 0.5 CoO | -0.040 |
| MoO_2_ | 0.6667 LiCoO_2_ + 0.3333 MoO_2_ → 0.6667 CoO + 0.3333 Li_2_MoO_4_ | -0.088 |
| Na_2_O | 0.6 Na_2_O + 0.4 LiCoO_2_ → 0.4 Na_3_CoO_3_ + 0.2 Li2O | -0.074 |
| Nb_2_O_5_ | 0.4286 LiCoO_2_ + 0.5714 Nb_2_O_5_ → 0.2857 LiNb_3_O_8_ + 0.1429 Nb_2_CoO_6_ + 0.1429 Li(CoO_2_)_2_ | -0.005 |
| NiO | LiCoO_2_ + NiO → No Reaction | 0.000 |
| PdO | LiCoO_2_ + PdO → No Reaction | 0.000 |
| Rb_2_O | 0.4167 LiCoO_2_ + 0.5833 Rb_2_O → 0.125 Rb_6_Co_2_O_7_ + 0.08333 Rb_5_(CoO_2_)_2_ + 0.2083 Li_2_O | -0.107 |
| Sc_2_O_3_ | LiCoO_2_ + Sc_2_O_3_ → No Reaction | 0.000 |
| SnO_2_ | LiCoO_2_ + SnO_2_ → No Reaction | 0.000 |
| SrO | LiCoO_2_ + SrO → No Reaction | 0.000 |
| TiO_2_ | LiCoO_2_ + TiO_2_ → No Reaction | 0.000 |
| V_2_O_5_ | 0.4 V_2_O_5_ + 0.6 LiCoO_2_ → 0.2 V_2_CoO_6_ + 0.4 LiVO_3_ + 0.2 Li(CoO_2_)_2_ | -0.035 |
| Y_2_O_3_ | LiCoO_2_ + Y_2_O_3_ → No Reaction | 0.000 |
| ZnO | LiCoO_2_ + ZnO → No Reaction | 0.000 |
| ZrO_2_ | LiCoO_2_ + ZrO_2_ → No Reaction | 0.000 |
| SiO_2_ | LiCoO_2_ + SiO_2_ → No Reaction | 0.000 |

**Table S4.** DFT-calculated reactions and thermodynamic reaction energies at LiMnO_2_ || Oxide interfaces for all oxides considered in Figure 2.

| **Oxide** | **LiMnO_2_ \|\| Oxide Reaction** | $\boldsymbol{\Delta}$**E (eV/atom)** |
| --- | --- | --- |
| Ag_2_O | 0.3 Ag_2_O + 0.7 LiMn_2_O_4_ → 0.6 MnAgO_2_ + 0.1 Li_5_Mn_7_O_16_ + 0.1 Li_2_MnO_3_ | -0.020 |
| Al_2_O_3_ | 0.1525 LiMn_2_O_4_ + 0.8475 Al2O3 → 0.1017 Mn3O4 + 0.08475 Al11O18 + 0.1525 LiAl5O8 | -0.024 |
| BaO | 0.5714 BaO + 0.4286 LiMn_2_O_4_ → 0.4286 LiMnO_2_ + 0.1429 Ba_4_Mn_3_O_10_ | -0.110 |
| CaO | 0.4545 CaO + 0.5455 LiMn_2_O_4_ → 0.2727 Li_2_MnO_3_ + 0.2727 CaMn_2_O_4_ + 0.09091 Ca_2_Mn_3_O_8_ | -0.041 |
| CdO | 0.4545 CdO + 0.5455 LiMn_2_O_4_ → 0.2727 Mn_2_CdO_4_ + 0.09091 Mn_3_Cd_2_O_8_ + 0.2727 Li_2_MnO_3_ | -0.014 |
| CoO | 0.6667 CoO + 0.3333 LiMn_2_O_4_ → 0.3333 Mn_2_CoO_4_ + 0.3333 LiCoO_2_ | -0.026 |
| Cr_2_O_3_ | 0.3333 Cr_2_O_3_ + 0.6667 LiMn_2_O_4_ → 0.3333 Mn_2_O_3_ + 0.6667 LiMnCrO_4_ | -0.024 |
| Cu_2_O | 0.5 Cu_2_O + 0.5 LiMn_2_O_4_ → 0.75 MnCuO_2_ + 0.25 Li_2_MnO_3_ + 0.25 CuO | -0.031 |
| FeO | 0.6667 FeO + 0.3333 LiMn_2_O_4_ → 0.3333 LiMnO_2_ + 0.3333 Mn(FeO_2_)_2_ | -0.099 |
| Ga_2_O_3_ | 0.2941 Ga_2_O_3_ + 0.7059 LiMn_2_O_4_ → 0.2941 Mn_2_O_3_ + 0.1176 Li_5_Mn_7_O_16_ + 0.1176 LiGa_5_O_8_ | -0.004 |
| In_2_O_3_ | LiMn_2_O_4_ + In_2_O_3_ → No Reaction | 0.000 |
| K_2_O | 0.6939 K_2_O + 0.3061 LiMn_2_O_4_ → 0.02041 K_11_LiMn_4_O_16_ + 0.3878 K_3_MnO_3_ + 0.1429 Li_2_MnO_3_ | -0.169 |
| Li_2_O | 0.5 Li_2_O + 0.5 LiMn_2_O_4_ → 0.5 LiMnO_2_ + 0.5 Li_2_MnO_3_ | -0.106 |
| MgO | 0.4286 MgO + 0.5714 LiMn_2_O_4_ → 0.2857 MgMn_2_O_4_ + 0.1429 Li_2_MgMn_3_O8 + 0.1429 Li_2_MnO_3_ | -0.020 |
| MnO | 0.6667 MnO + 0.3333 LiMn_2_O_4_ → 0.3333 LiMnO_2_ + 0.3333 Mn_3_O_4_ | -0.054 |
| MoO_2_ | 0.5 LiMn_2_O_4_ + 0.5 MoO_2_ → 0.25 MnMoO_4_ + 0.25 Li_2_MoO_4_ + 0.25 Mn_3_O_4_ | -0.079 |
| Na_2_O | 0.5 Na_2_O + 0.5 LiMn_2_O_4_ → 0.25 Li_2_MnO_3_ + 0.25 Na_2_MnO_3_ + 0.5 NaMnO_2_ | -0.160 |
| Nb_2_O_5_ | 0.1667 Nb_2_O_5_ + 0.8333 LiMn_2_O_4_ → 0.1667 MnNb_2_O_6_ + 0.1667 Li_5_Mn_7_O_16_ + 0.1667 Mn_2_O_3_ | -0.004 |
| NiO | 0.7273 LiMn_2_O_4_ + 0.2727 NiO → 0.2727 Li_2_Mn_3_NiO_8_ + 0.09091 Li_2_MnO_3_ + 0.1818 Mn_3_O_4_ | -0.010 |
| PdO | LiMn_2_O_4_ + PdO → No Reaction | 0.000 |
| Rb_2_O | 0.3158 LiMn_2_O_4_ + 0.6842 Rb_2_O → 0.1579 Li_2_MnO_3_ + 0.4211 Rb_3_MnO_3_ + 0.05263 Rb_2_MnO_4_ | -0.160 |
| Sc_2_O_3_ | LiMn_2_O_4_ + Sc_2_O_3_ → No Reaction | 0.000 |
| SnO_2_ | LiMn_2_O_4_ + SnO_2_ → No Reaction | 0.000 |
| SrO | 0.7474 SrO + 0.2526 LiMn_2_O_4_ → 0.01053 Sr_7_Mn_4_O_15_ + 0.04211 Sr_16_Mn_8_O_29_ + 0.1263 Li_2_MnO_3_ | -0.076 |
| TiO_2_ | LiMn_2_O_4_ + TiO_2_ → No Reaction | 0.000 |
| V_2_O_5_ | 0.5 V_2_O_5_ + 0.5 LiMn_2_O_4_ → 0.5 LiVO_3_ + 0.25 MnV_2_O_6_ + 0.75 MnO_2_ | -0.019 |
| Y_2_O_3_ | 0.3333 Y_2_O_3_ + 0.6667 LiMn_2_O_4_ → 0.3333 YMnO_3_ + 0.3333 YMn_2_O_5_ + 0.3333 Li_2_MnO_3_ | -0.030 |
| ZnO | 0.4286 ZnO + 0.5714 LiMn_2_O_4_ → 0.1429 Li_2_Mn_3_ZnO_8_ + 0.2857 Mn_2_ZnO_4_ + 0.1429 Li_2_MnO_3_ | -0.013 |
| ZrO_2_ | LiMn_2_O_4_ + ZrO_2_ → No Reaction | 0.000 |
| SiO_2_ | 0.1552 SiO_2_ + 0.8448 LiMn_2_O_4_ → 0.1552 Li_5_Mn_7_O_16_ + 0.08621 Mn_7_SiO_12_ + 0.03448 Li_2_Si_2_O_5_ | -0.004 |

**Considering Three-Phase Reactions**

A representative 3-phase contact formed between LPSCl electrolyte, Li anode, and Al2O3 coating is considered below. Instead of directly modeling the triple junction, we studied the interactions between various reaction products formed at the LPSCl || Al_2_O_3_ interface (Table 1 of the manuscript) and Li || Al_2_O_3_ interface (Table 2 of the manuscript). The reactions at these two separate interfaces are listed below:

**Reaction**  **eV/atom**

0.2 Li_6_PS_5_Cl + 0.8 Al_2_O_3_ → 0.2 Li_3_PS_4_ + 0.2 LiCl + 0.1 LiAlS_2_ + 0.3 LiAl_5_O_8_ -0.044

0.6667 Li + 0.3333 Al_2_O_3_ → 0.1667 LiAl + 0.5 LiAlO_2_ -0.220

They contain 4 and 2 reaction products respectively. There are 8 additional second-order reactions between the prior reaction products:

**Reaction**  **eV/atom**

0.3333 Li_3_PS_4_ + 0.6667 LiAl → 0.3333 LiAlS_2_ + 0.3333 AlP + 0.6667 Li_2_S -0.516

0.9091 LiAlO_2_ + 0.09091 Li_3_PS_4_ → 0.1818 LiAl_5_O_8_ + 0.3636 Li_2_S + 0.09091 Li_3_PO_4_ -0.023

LiCl + LiAl → No reaction 0.000

LiCl + LiAlO_2_ → No reaction 0.000

0.25 LiAlS_2_ + 0.75 LiAl → 0.5 Li_2_S + Al -0.183

0.2 LiAlS_2_ + 0.8 LiAlO_2_ → 0.2 LiAl_5_O_8_ + 0.4 Li_2_S -0.024

0.25 LiAl_5_O_8_ + 0.75 LiAl → LiAlO_2_ + Al -0.068

LiAl_5_O_8_ + LiAlO_2_ → No reaction 0.000

Across these 8 reactions, there are six distinct compounds. Four of these compounds (LiAlS_2_, Li_2_S, LiAl_5_O_8_, Li_3_PO_4_) have already been investigated in the manuscript since they are formed at the various 2-phase contacts across all modeled oxides. They all support fast Li^+^ conduction. The fifth product is Al, which is metallic and known to alloy with Li, which should improve interfacial wetting. The sixth compound is AlP, which is semiconducting. We note that 3-phase contact may suppress the formation of some of these compounds; however, overall no significant changes to the conclusions drawn from our 2-phase analysis arise when considering 3-phase reactions, at least for Al_2_O_3_-based coatings. This may not hold true for every system, however, and this is an important area for future study.

**Table S5.** DFT-calculated bandgaps calculated for all oxides considered in Figure 3a.

| **Oxide** | **HSE bulk bandgap (eV)** |
| --- | --- |
| Ag_2_O | 1.50 |
| Al_2_O_3_ | 8.80 |
| BaO | 3.68 |
| CaO | 5.80 |
| CdO | 1.30 |
| CoO | 2.41 |
| Cr_2_O_3_ | 4.94 |
| Cu_2_O | 2.45 |
| FeO | 2.27 |
| Ga_2_O_3_ | 4.65 |
| In_2_O_3_ | 3.20 |
| K_2_O | 3.50 |
| Li_2_O | 7.15 |
| MgO | 7.16 |
| MnO | 2.33 |
| MoO_2_ | 0.37 |
| Na_2_O | 6.16 |
| Nb_2_O_5_ | 4.73 |
| NiO | 3.96 |
| PdO | 2.06 |
| Rb_2_O | 3.04 |
| Sc_2_O_3_ | 6.26 |
| SnO_2_ | 3.56 |
| SrO | 5.20 |
| TiO_2_ | 4.26 |
| V_2_O_5_ | 4.05 |
| Y_2_O_3_ | 6.23 |
| ZnO | 3.00 |
| ZrO_2_ | 5.88 |
| SiO_2_ | 8.12 |

**Table S6.** Li migration barriers and bandgaps of reaction products at the LPSCl || Oxide interface, obtained from the Materials Project (using the pseudopotential of Perdew-Burke-Ernzerhof). Oxides are listed in the order of increasing reactivities at the LPSCl || oxide interface. Only products containing the primary element (element in the oxide) are listed. Other Li-containing products such as LiCl, Li_3_PO_4_, and Li_3_PS_4_ are known fast lithium conductors. The PBE bandgap of LPSCl is 2.14 eV.

| **Oxide** | **Reaction Product(s)** | **Li Migration Barrier (eV)** | **Bandgap (eV)** |
| --- | --- | --- | --- |
| SiO_2_ | N/A | N/A | N/A |
| AlO_3_ | LiAlS_2_  LiAl_5_O_8_ | 0.28  0.28 | 4.11  5.25 |
| ZrO_2_ | ZrS_2_ | 0.16 | 1.04 |
| MgO | MgS | 1.15 | 2.76 |
| TiO_2_ | TiS_3_  Li_4_TiS_4_  Li(TiS_2_)_2_ | 0.21  0.30  0.35 | 0.23  2.37  0.00 |
| Sc_2_O_3_ | LiScS_2_ | 0.54 | 1.49 |
| Y_2_O_3_ | LiYS_2_ | 0.59 | 1.92 |
| Nb_2_O_5_ | NbS_3_  Li_5_(NbS_2_)_7_ | 0.10  0.39 | 0.00  0.00 |
| MnO | MnS  Li_6_MnS_4_ | 0.51  0.24 | 0.00  1.55 |
| Cr_2_O_3_ | LiCrS_2_ | 0.68 | 0.65 |
| Li_2_O | Li_2_S | 0.25 | 3.39 |
| SnO_2_ | SnS_2_ | – | 1.51 |
| Ga_2_O_3_ | LiGaS_2_ | – | 2.96 |
| CaO | CaS | – | 2.38 |
| In_2_O_3_ | LiInS_2_ | – | 2.22 |
| ZnO | ZnS | 0.54 | 2.02 |
| FeO | FeS | – | 0.00 |
| Cu_2_O | Cu_7_S_4_ | – | 0.00 |
| SrO | SrS | – | 2.50 |
| V_2_O_5_ | VS_2_ | – | 0.00 |
| NiO | Ni_9_S_8_ | – | 0.00 |
| CoO | Co_9_S_8_ | – | 0.00 |
| MoO_2_ | MoS_2_ | – | 1.20 |
| Na_2_O | Na_2_S | – | 2.44 |
| CdO | CdS | – | 1.13 |
| BaO | BaS | – | 2.15 |
| Ag_2_O | Ag_2_S | – | 0.00 |
| K_2_O | K_2_S | – | 2.32 |
| Rb_2_O | Rb_2_S | – | 1.96 |
| PdO | PdS | – | 0.00 |

**Table S7.** Li migration barriers and bandgaps of non-metallic reaction products at the Li || Oxide interface, obtained from the Materials Project (using the pseudopotential of Perdew-Burke-Ernzerhof). Oxides are listed in the order of increasing reactivities at the Li || oxide interface. The PBE bandgap of LPSCl is 2.14 eV.

| **Oxide** | **Product(s)** | **Li Migration Barrier (eV)** | **Bandgap (eV)** |
| --- | --- | --- | --- |
| Y_2_O_3_ | N/A | N/A | N/A |
| CaO | N/A | N/A | N/A |
| Li_2_O | N/A | N/A | N/A |
| SrO | Li_2_O | 0.19 | 4.90 |
| Sc_2_O_3_ | LiScO_2_ | 0.61 | 3.90 |
| MgO | Li_2_O | 0.19 | 4.90 |
| BaO | Li_2_O | 0.19 | 4.90 |
| ZrO_2_ | Zr_3_O  Li_6_Zr_2_O_7_ | 1.59  0.56 | 0.00  3.84 |
| Al_2_O_3_ | LiAlO_2_ | 0.53 | 6.12 |
| TiO_2_ | Ti_2_O  Li_4_TiO_4_ | 0.00  0.66 | 0.00  4.56 |
| Na_2_O | Li_2_O | 0.19 | 4.90 |
| SiO_2_ | Li_4_SiO_4_ | 0.26 | 4.78 |
| K_2_O | Li_2_O | 0.19 | 4.90 |
| Rb_2_O | Li_2_O | 0.19 | 4.90 |
| MnO | Li_2_O | 0.19 | 4.90 |
| Nb_2_O_5_ | LiNbO_2_ | 0.51 | 1.58 |
| Cr_2_O_3_ | Li_2_O | 0.19 | 4.90 |
| ZnO | Li_2_O | 0.19 | 4.90 |


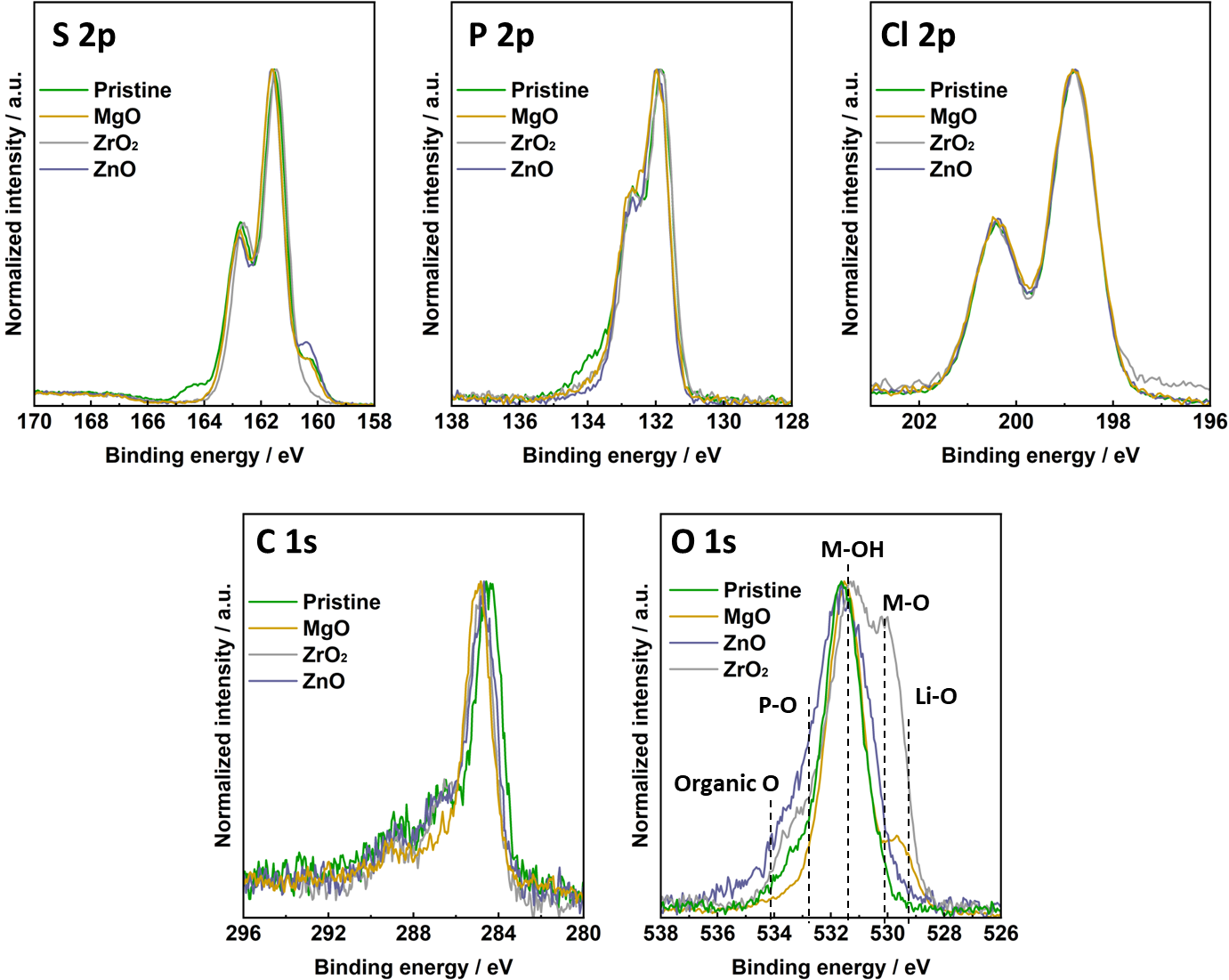


**Figure S1. Comparing core level spectra of underlying LPSCl with pristine surface.** The intensities are normalized without background subtraction. The spectra are collected on the pellet pressed from the coated powders.


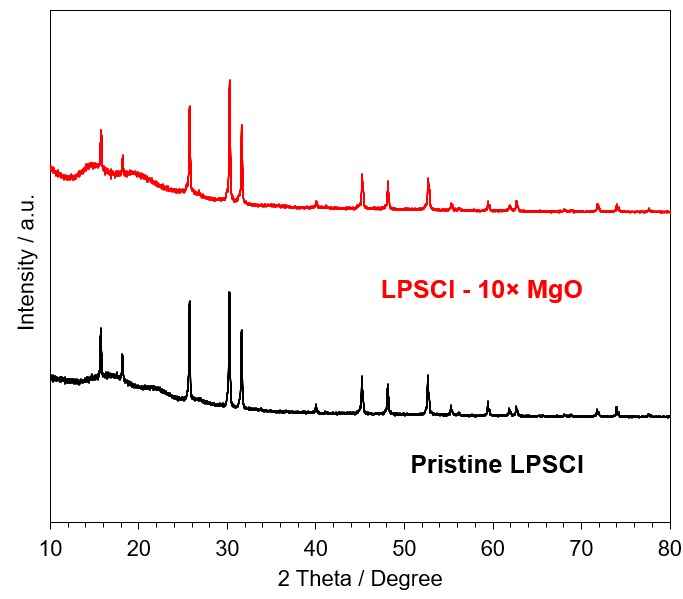


**Figure S2.** X-ray diffraction of as-synthesized materials as well as 10x MgO coated LPSCl.

**Table S8.** XPS peak fitting parameters used to determine the chemical composition of LPSCl with ALD layers.

| **ALD Chemistry** | **Core Level** | **Components** | **BE (eV)** | **FWHM (eV)** | **Composition (at%)** |
| --- | --- | --- | --- | --- | --- |
| **Pristine**  **LPSCl** | S 2p | PS_4_^3-^ | 161.5 / 162.6 | 0.83 | 64.1 |
|  |  | M-S | 160.3 / 161.4 | 0.89 | 11.2 |
|  |  | P_2_S_x_ | 163.1 / 164.3 | 0.79 | 8.5 |
|  |  | PO_x_S_y_ | 161.8 / 163.0 | 0.82 | 15.6 |
|  | P 2p | PS_4_^3-^ | 131.8 / 132.7 | 0.77 | 82.4 |
|  |  | PO_x_S_y_ | 132.9 / 133.8 | 1.01 | 8.8 |
|  |  | P-O | 133.5 / 134.4 | 1.09 | 8.8 |
|  | Cl 2p | LiCl | 198.8 / 200.4 | 1.07 | 100.0 |
| **MgO** | S 2p | PS_4_^3-^ | 161.6 / 162.7 | 0.82 | 63.7 |
|  |  | M-S | 160.4 / 161.5 | 0.95 | 12.6 |
|  |  | PO_x_S_y_ | 161.9 / 163.0 | 0.88 | 23.8 |
|  | P 2p | PS_4_^3-^ | 132.0 / 132.8 | 0.81 | 90.0 |
|  |  | PO_x_S_y_ | 132.6 / 133.5 | 1.02 | 4.9 |
|  |  | P-O | 133.5 / 134.4 | 1.29 | 5.2 |
|  | Cl 2p | LiCl | 198.8 / 200.4 | 1.06 | 100.0 |
|  | Mg 2p | LiMgO | 49.2 | 1.00 | 65.6 |
|  |  | Mg-OH | 49.9 | 1.07 | 33.4 |
|  |  | Mg-O / Mg-S | 50.8 | 1.00 | 1.1 |
| **ZnO** | S 2p | PS_4_^3-^ | 161.6 / 162.7 | 0.82 | 62.2 |
|  |  | M-S | 160.3 / 161.5 | 0.90 | 16.2 |
|  |  | PO_x_S_y_ | 161.9 / 163.0 | 0.92 | 20.8 |
|  | P 2p | PS_4_^3-^ | 131.9 / 132.8 | 0.75 | 89.3 |
|  |  | PO_x_S_y_ | 132.5 / 133.4 | 0.99 | 6.9 |
|  |  | P-O | 133.5 / 134.4 | 1.29 | 3.8 |
|  | Cl 2p | LiCl | 198.8 / 200.4 | 1.07 | 100.0 |
| **ZrO_2_** | S 2p | PS_4_^3-^ | 161.5 / 162.6 | 0.87 | 86.9 |
|  |  | M-S | 160.3 / 161.5 | 0.86 | 2.8 |
|  |  | PO_x_S_y_ | 162.0 / 163.2 | 1.2 | 9.6 |
|  | P 2p | PS_4_^3-^ | 131.8 / 132.7 | 0.74 | 83.0 |
|  |  | PO_x_S_y_ | 132.6 / 133.5 | 1.29 | 16.0 |
|  |  | P-O | 133.8 / 134.6 | 1.09 | 1.0 |
|  | Cl 2p | LiCl | 198.8 / 200.4 | 1.09 | 100.0 |
|  | Zr 3d | ZrO_2_ | 182.0 / 184.4 | 1.13 | 86.3 |
|  |  | Li_6_Zr_2_O_7_ | 179.7 / 182.1 | 1.35 | 6.7 |
|  |  | Zr_3_O | 178.2 / 180.6 | 1.36 | 7.1 |


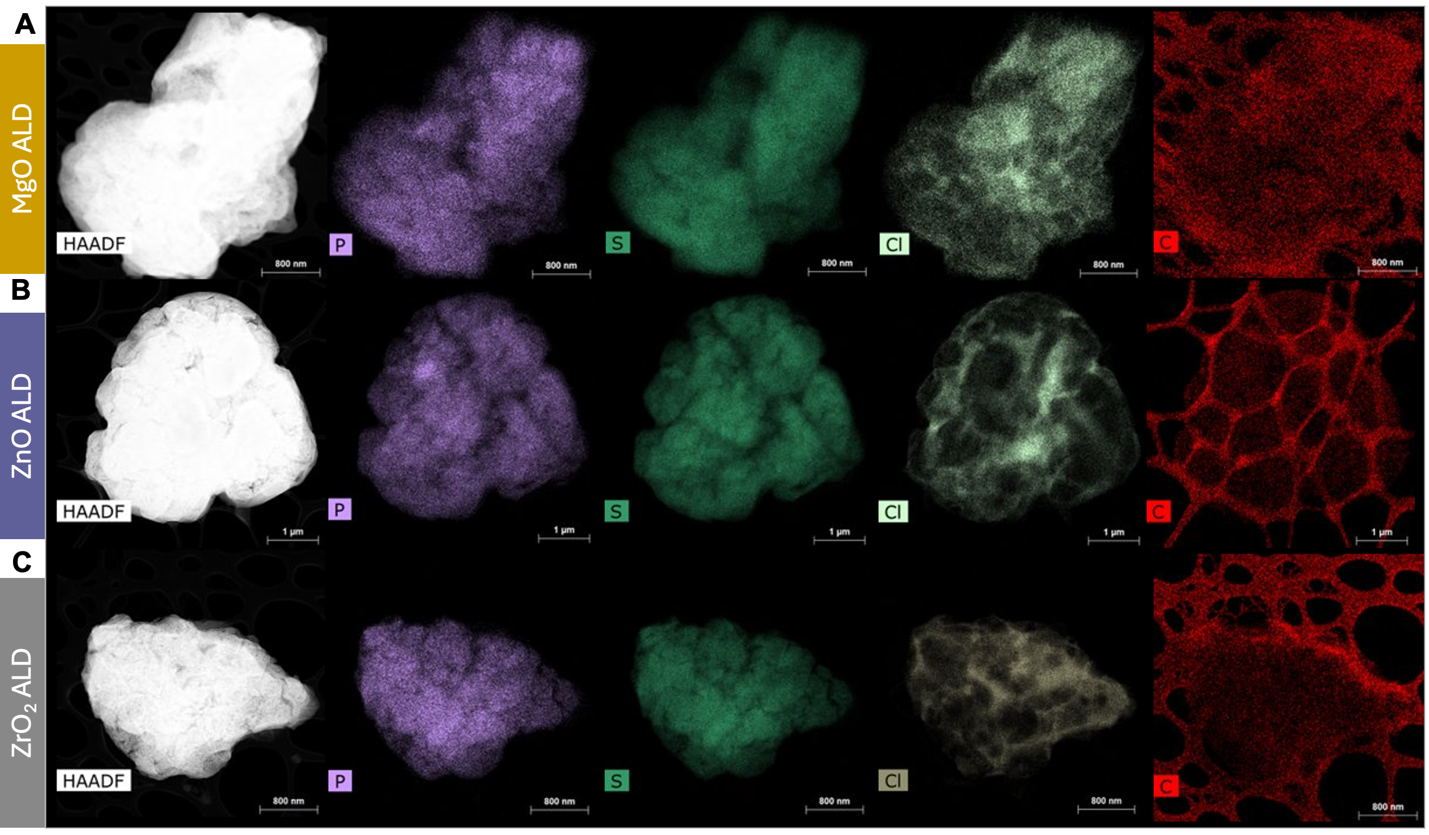


**Figure S3. Extended STEM-EDS Mapping of ALD coated LPSCl particles.** EDS maps for P, S, Cl and C are shown for LPSCl coated with **(A)** MgO, **(B)** ZnO and **(C)** ZrO_2_, respectively.

EDS maps showing the distribution of P and S are shown in **Figure S2,** which generally scale in intensity with the particle thickness as indicated in the HAADF images. Also shown in the same figure are Cl maps which show some segregation between LPSCl particles, this, however, is likely a sublimation artifact caused of the high vacuum environment (10^-7^-10^-8^ Torr) present in the TEM column. Elemental maps for carbon are also shown; of the three chemistries, MgO coated LPSCl show the most uniform C coverage with increased C presence near the edges of the particles suggesting the MgO coating incorporates some organic species.


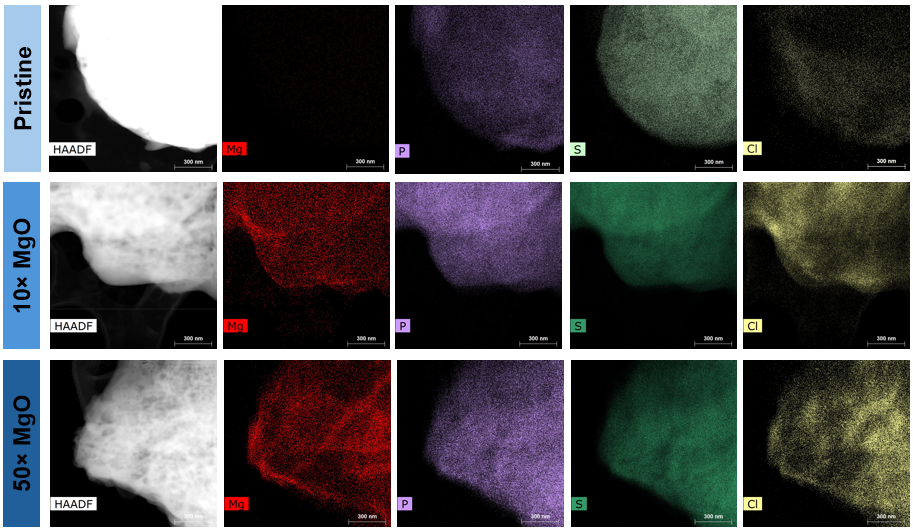


**Figure S4.** STEM/EDS analysis of LPSCl particles before and after MgO ALD coating. The pristine LPSCl particles show no evidence of MgO coating. After 10× MgO ALD cycles, a uniform and conformal MgO layer is observed, with no noticeable change in particle size. After 50× MgO ALD cycles, the MgO content increases while the coating remains uniform and the particle size remains consistent. These results indicate that MgO coating via ALD does not alter the core morphology or size of the LPSCl particles across the tested deposition cycles.

STEM/EDS imaging of pristine, 10×, and 50× MgO-coated Li₆PS₅Cl particles (Figure S4) provides strong evidence of both coating conformity and surface coverage. Pristine (uncoated) LPSCl particles show no detectable Mg signal, confirming the absence of any native Mg-containing layers before ALD. After 10× MgO ALD cycles, the EDS maps reveal a thin but continuous Mg signal localized at particle edges, consistent with a conformal and complete surface coating. No patchy or uncoated regions were observed, and this uniformity was consistent across multiple particles analyzed.

After 50× MgO ALD cycles, the Mg signal intensity increases significantly, confirming higher MgO content, while the coating remains continuous and uniformly distributed around the particles. The particle size remains consistent with the pristine and 10× samples, indicating conformal growth without agglomeration. These results, supported by the absence of new phases in XRD patterns, confirm that the MgO coating remains surface-localized and does not alter the bulk argyrodite phase. We have included additional STEM/EDS images in the Supporting Information to illustrate coating uniformity and completeness more comprehensively. Overall, these findings confirm that MgO coatings produced via ALD are uniform, conformal, and complete, even at relatively low cycle numbers, demonstrating the efficacy of this surface modification approach for sulfide-based solid electrolytes.

**
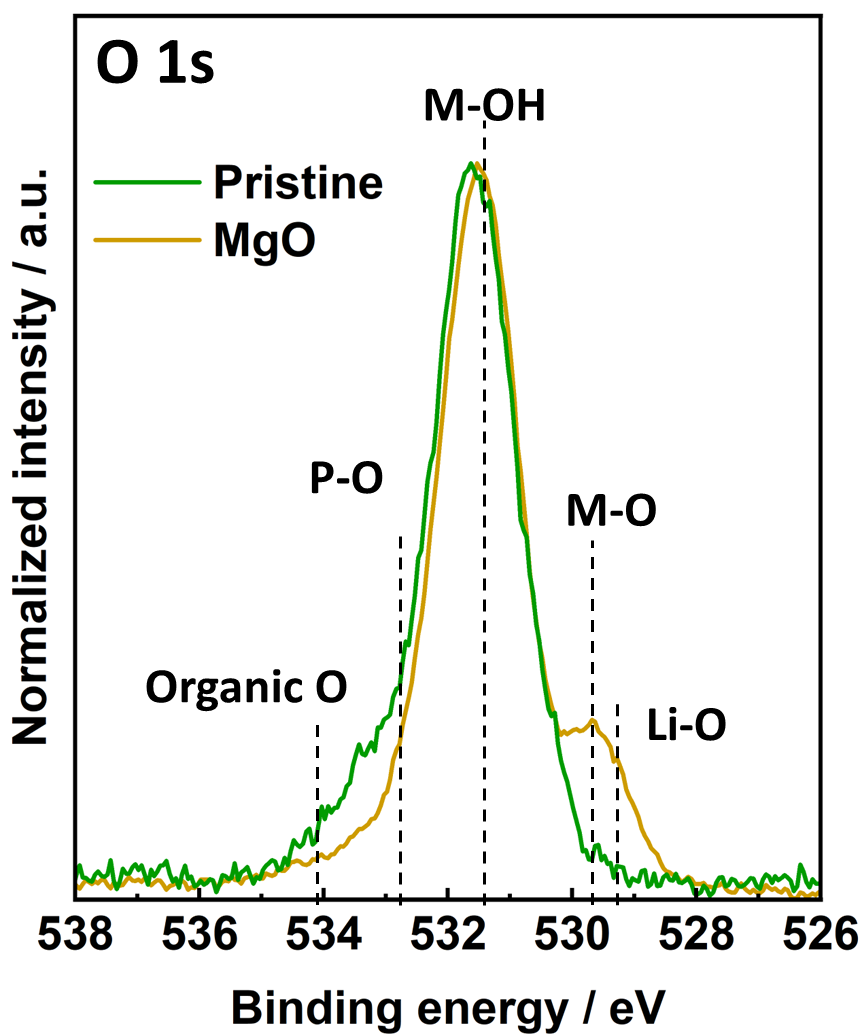
**

**Figure S5.** Comparing O 1s core levels of the MgO ALD and the pristine LPSCl.


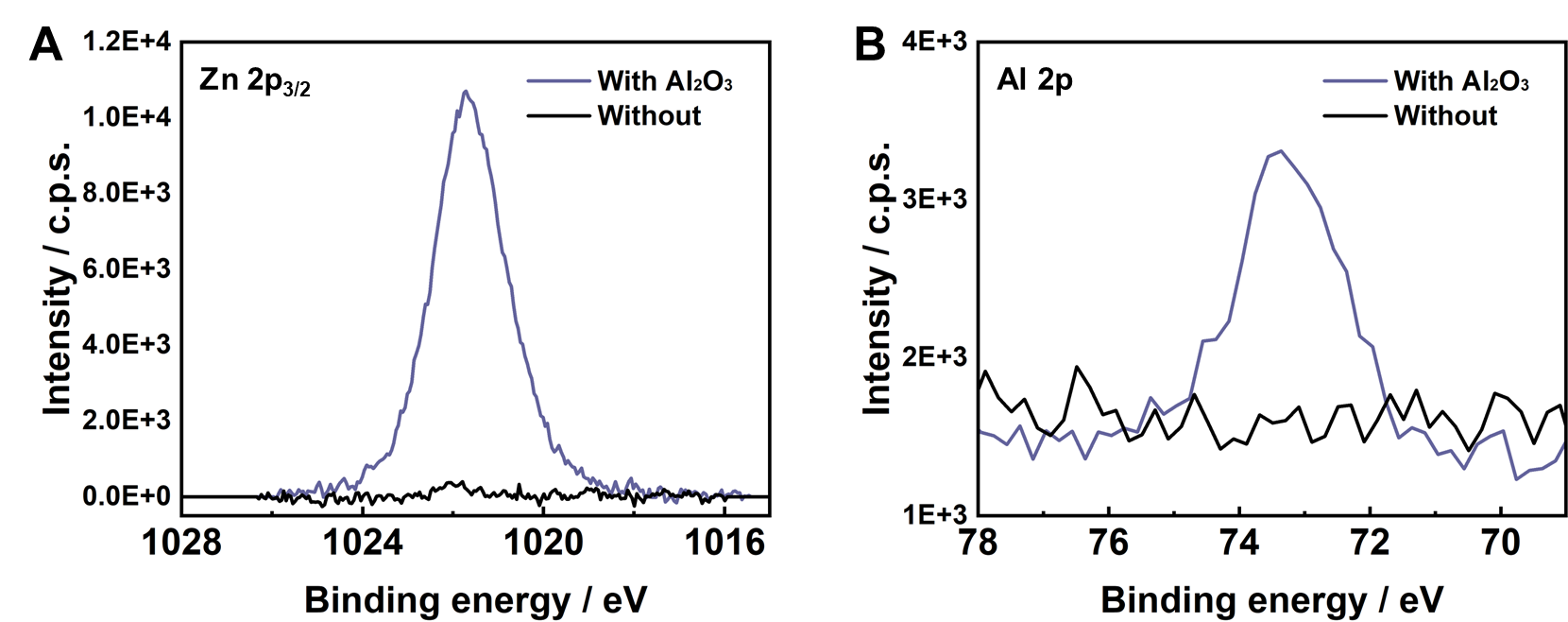


**Figure S6. Comparing core levels on the ZnO ALD LPSCl.** (A) background subtracted Zn 2p_3/2_ core level spectra and (B) Al 2p core level spectra from survey scan without background subtract. The ZnO ALD LPSCl with and without Al_2_O_3_ seed layer is measured on powders.


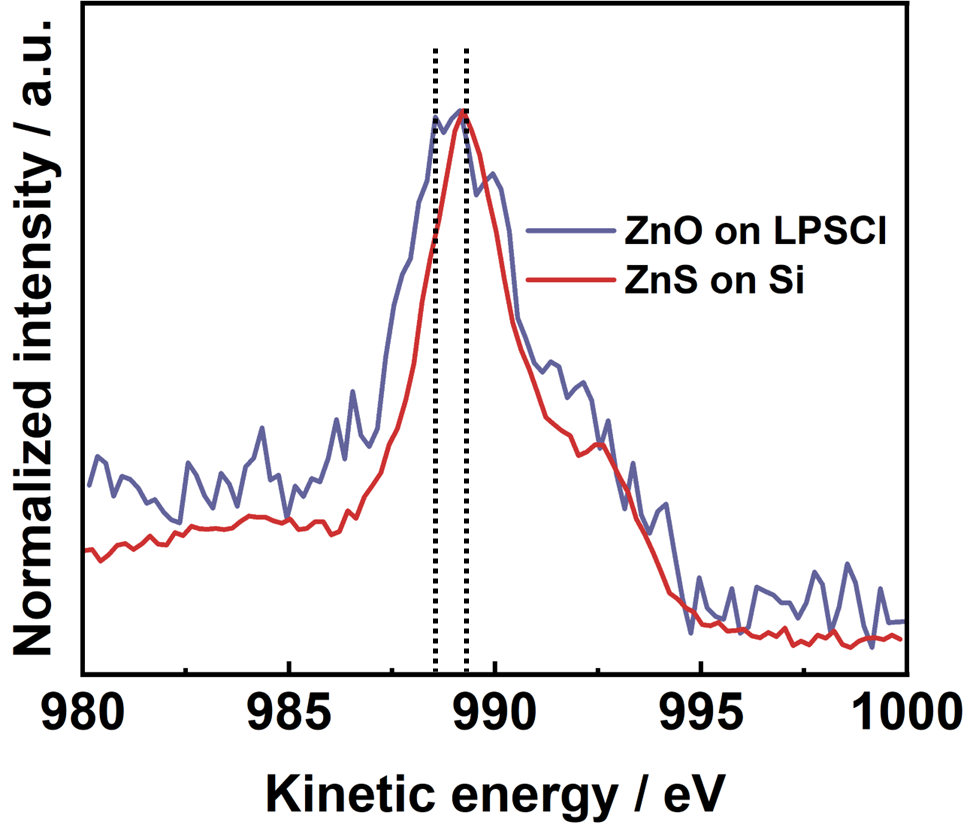


**Figure S7.** Zn LMM Auger spectra of ZnO ALD on LPSCl with Al_2_O_3_ seed layer and ZnS on Si wafer.

As there is only a marginal difference of binding energy between ZnO and ZnS in Zn 2p_3/2_, we instead compare the Zn LMM Auger feature from XPS of ZnO-coated LPSCl with ZnS ALD grown on Si wafer (Figure S#3). We observe a strong signal at 989 eV (kinetic energy) on both surfaces, which is ascribed to ZnS, while the ZnO ALD LPSCl also exhibits signal at lower kinetic energy (~988 eV) that corresponds to ZnO^1^. This indicates that the increased metal sulfide intensity of the metal sulfide doublet is derived from ZnS which is probably formed during ALD process in addition to the signal of residue of Li_2_S during powder synthesis.

_
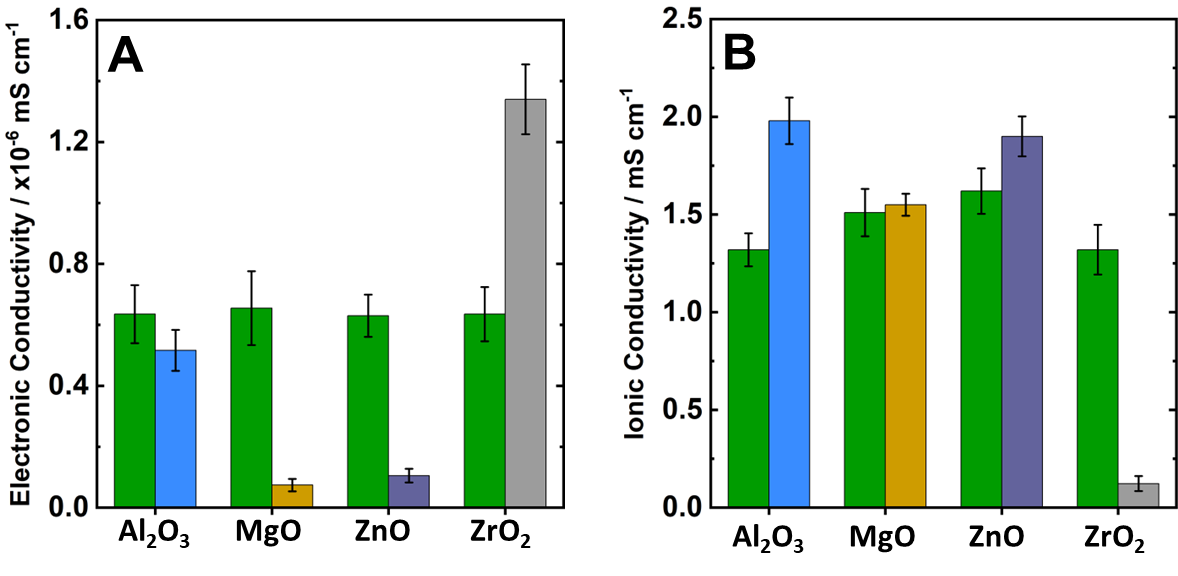
_

**Figure S8. Electrochemical properties of ALD LPSCl pellets.** (A) Electronic and (B) Ionic conductivities. ZnO ALD has 1 cycle Al_2_O_3_ ALD seed layer on the pristine LPSCl.


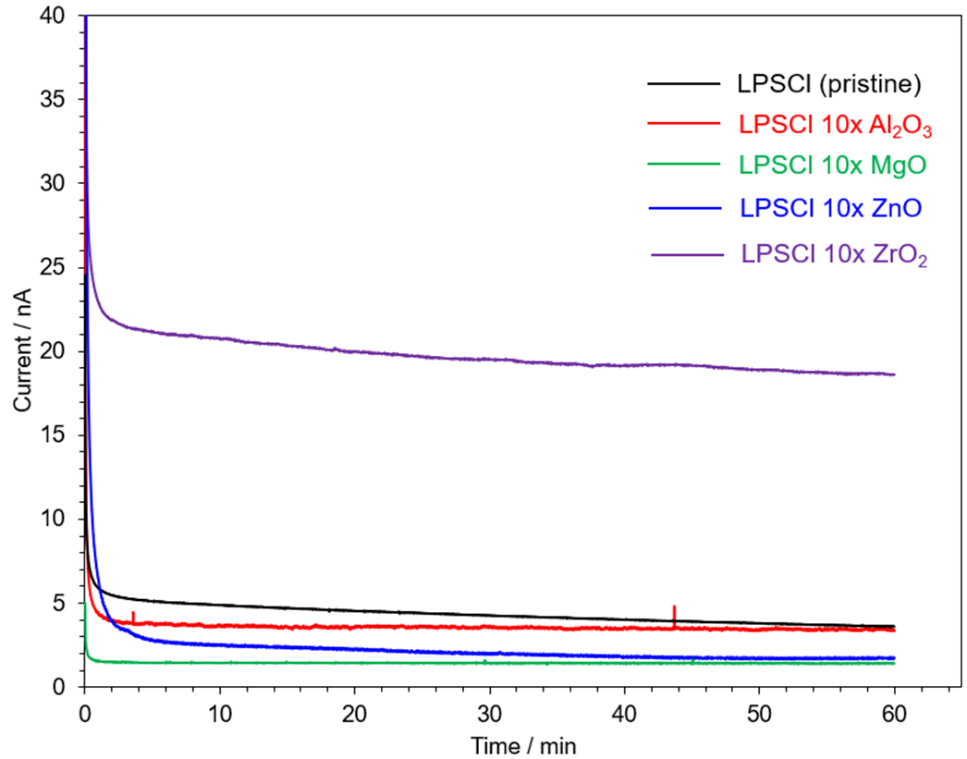


**Figure S9.** DC electronic conductivity at 25°C for Li₆PS₅Cl pellets pressed from powders coated with 10× ALD cycles of Al₂O₃, MgO, ZnO, and ZrO₂, compared to the pristine (uncoated) material.


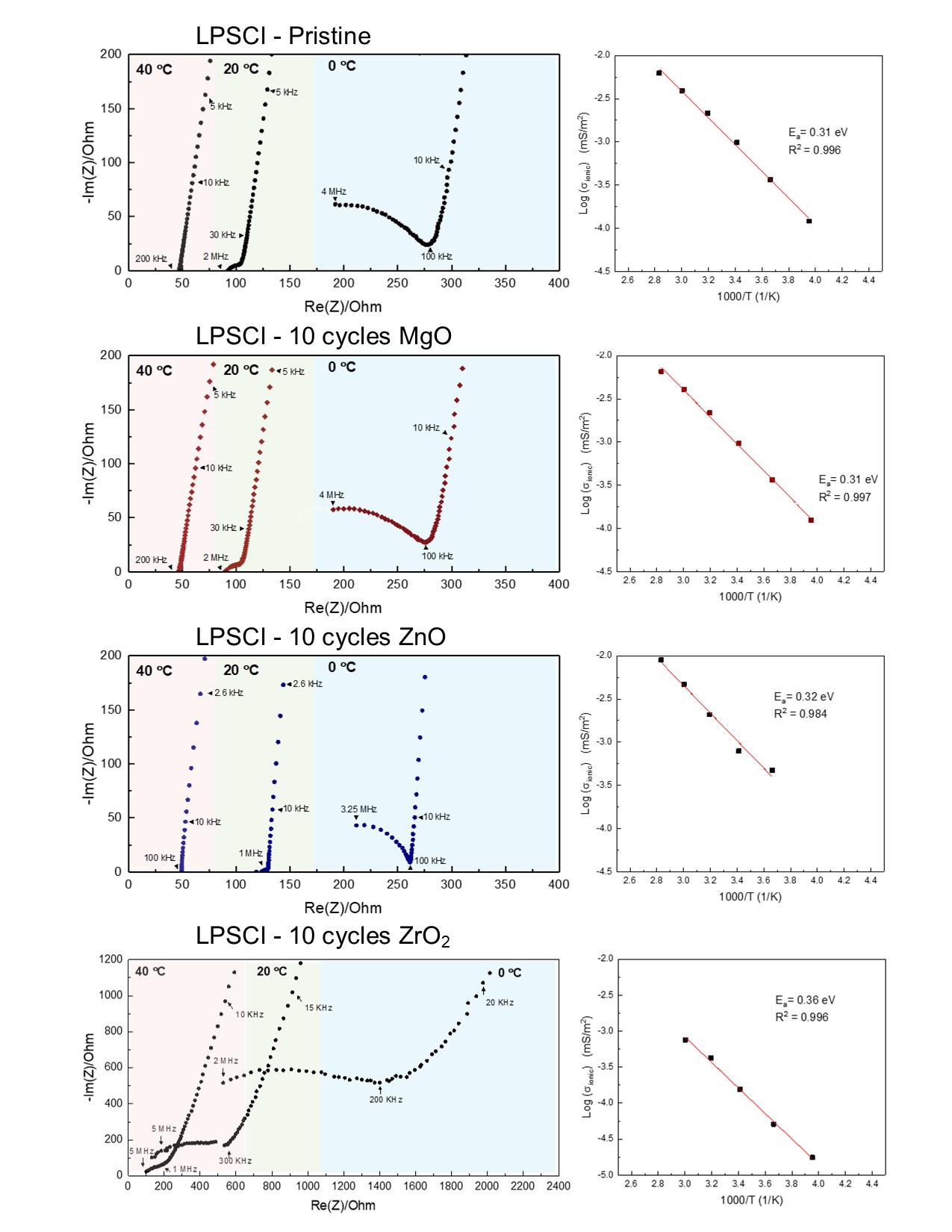


**Figure S10.** Temperature-dependent EIS (left) and Arrhenius activation energy (right) measurements of coated and uncoated LPSCl materials.

The impact of ALD coatings on the DC electronic conductivity of Li₆PS₅Cl is substantial and varies significantly depending on the coating material (Figure S9). MgO and ZnO coatings both lead to notable reductions in electronic conductivity—by nearly an order of magnitude—compared to the pristine material, with conductivity ratios of 0.11 and 0.17, respectively. This indicates that these coatings effectively suppress electronic transport, which may be beneficial for minimizing parasitic currents in solid-state battery applications. Interestingly, despite ZnO being intrinsically more conductive, the dramatic reduction in conductivity suggests that interfacial reactions between ZnO and Li₆PS₅Cl result in the formation of highly resistive species. A thinner alumina seed layer used during ZnO deposition has been shown to have minimal impact on conductivity, further confirming that the ZnO coating itself is responsible for the observed behavior. In contrast, Al₂O₃ coatings lead to only a modest decrease in electronic conductivity (ratio of 0.81), which aligns with expectations for a thin insulating layer. Notably, ZrO₂ coatings produce a twofold increase in electronic conductivity, likely due to the formation of interphases with narrower bandgaps than the original electrolyte, thereby enhancing electronic transport. These results highlight the critical role of interfacial chemistry in determining the electronic properties of coated solid electrolytes.

Nyquist plots derived from EIS measurements conducted at 0 °C, 20 °C, and 40 °C for pristine and ALD–coated samples, specifically with 10-cycle coatings of MgO, ZnO, and ZrO₂ are summarized in Figure S10. The frequency ranges are indicated in the plots, and the temperature dependence of the impedance arcs reflects changes in the ionic conductivity of the materials. The pristine and 10x MgO-coated samples show nearly identical activation energies of 0.31 eV, suggesting that the MgO coating does not significantly impede ion transport and may preserve the interfacial characteristics of the uncoated material. Similarly, the 10x ZnO-coated sample exhibits a slightly higher activation energy of 0.32 eV, indicating minimal additional resistance to ion migration through or across the coating. In contrast, the 10x ZrO₂-coated sample displays a noticeably higher activation energy of 0.36 eV. This increase may be attributed to the more insulating or blocking nature of the ZrO₂ coating, which could introduce additional barriers to charge transport at the interface or within the material, especially at lower temperatures. Overall, the Nyquist plots and extracted activation energies reveal that while MgO and ZnO coatings maintain favorable transport properties, ZrO₂ introduces increased resistance likely due to its more robust dielectric properties or greater thickness/density, thus slightly impeding the electrochemical performance.

**Table S9.** XPS peak fitting parameters used to determine the chemical composition of LPSCl after Li deposition.

| **ALD Chemistry** | **Core Level** | **Components** | **BE (eV)** | **FWHM (eV)** | **Composition (at%)** |
| --- | --- | --- | --- | --- | --- |
| **Pristine**  **LPSCl** | S 2p | PS_4_^3-^ | 161.4 / 162.6 | 0.81 | 8.8 |
|  |  | M-S | 160.2 / 161.3 | 0.91 | 83.3 |
|  |  | PO_x_S_y_ | 161.8 / 163.0 | 0.80 | 7.9 |
|  | P 2p | PS_4_^3-^ | 132.0 / 132.8 | 0.87 | 16.6 |
|  |  | PO_x_S_y_ | 132.7 / 1336 | 1.12 | 4.9 |
|  |  | Li_x_P | 127.1 / 128.0 | 1.13 | 26.3 |
|  |  | Li_3_P | 125.7 / 126.6 | 0.92 | 52.3 |
|  | Cl 2p | LiCl | 198.8 / 200.4 | 1.16 | 100.0 |
| **MgO** | S 2p | PS_4_^3-^ | 161.6 / 162.7 | 0.90 | 20.1 |
|  |  | M-S | 160.3 / 161.5 | 0.94 | 69.9 |
|  |  | PO_x_S_y_ | 161.8 / 163.0 | 0.80 | 10.0 |
|  | P 2p | PS_4_^3-^ | 132.0 / 132.9 | 0.74 | 28.3 |
|  |  | PO_x_S_y_ | 132.9 / 133.8 | 0.92 | 7.1 |
|  |  | Li_x-1_P | 129.4 / 130.2 | 1.32 | 8.9 |
|  |  | Li_x_P | 127.5 / 128.4 | 1.25 | 32.9 |
|  |  | Li_3_P | 125.9 / 126.8 | 0.96 | 22.8 |
|  | Cl 2p | LiCl | 198.8 / 200.4 | 1.12 | 100.0 |
|  | Mg 2p | Mg-Li | 48.2 | 1.1 | 47.3 |
|  |  | LiMgO | 49.0 | 0.93 | 48.8 |
|  |  | Mg-OH | 49.9 | 0.90 | 3.9 |

**
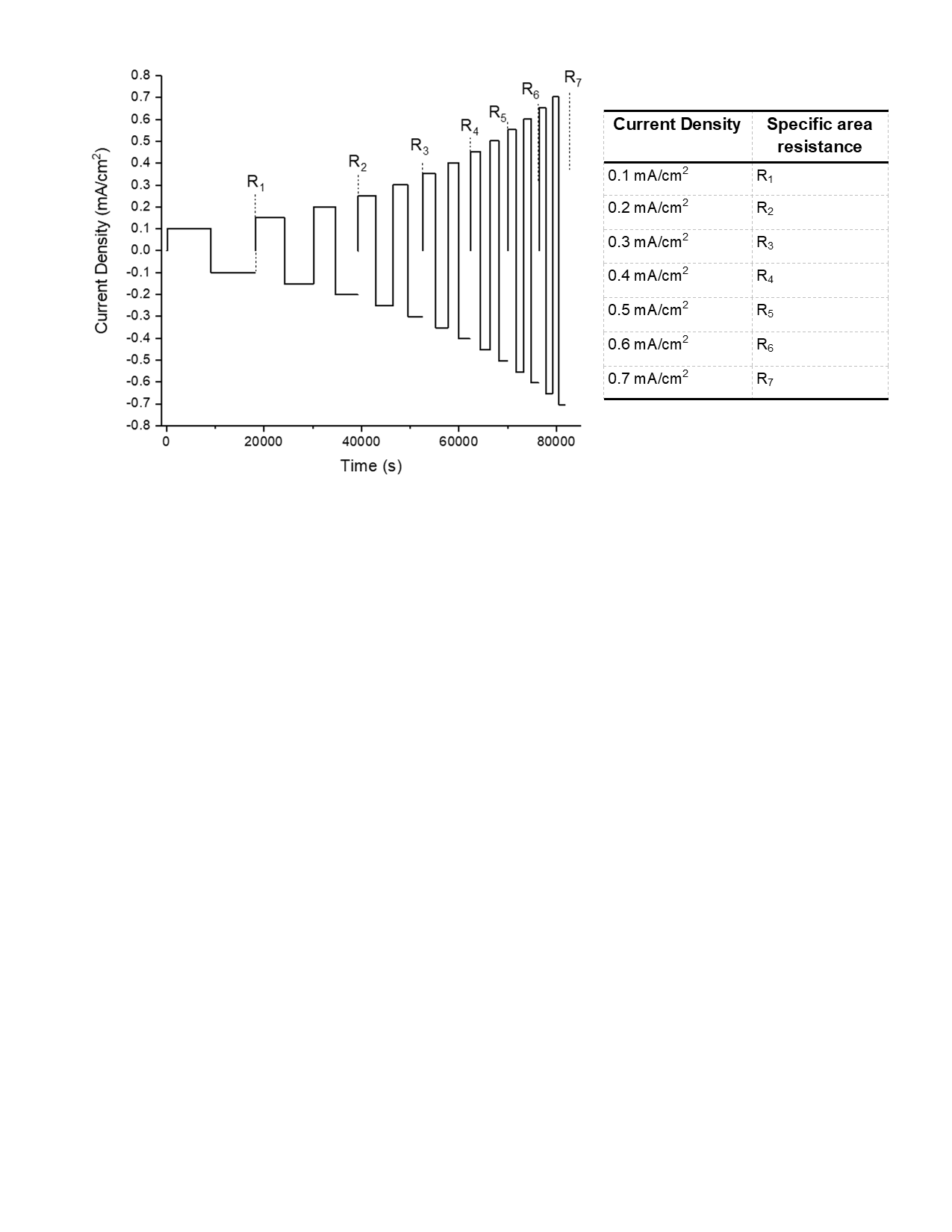
**

**Figure S11. Galvanostatic protocol for current density stability.** Beginning at 0.1 mA/cm^2^ the current density in increased by steps of 0.05mA/cm^2^ to a final value of 0.7 mA/cm^2^_._ As marked by the dashed line, an EIS measurement is acquired at each step of 0.1mA/cm^2^. The specific area resistance is then calculated from these values.

Figure S12a shows the baseline potential response from the uncoated LPSCl from the protocol detailed in Figure S11 and the corresponding resistance of the cell after the indicated applied current density. A clear decoupling between the applied current and the voltage response is observed between 0.4-0.5 mA/cm^2^, and in subsequent cycles a significant reduction in the area-specific resistance (ASR) occurs, suggesting that the cell has short circuited. The potential response from the MgO coated LPSCl largely resembles that of the uncoated material (Figure S8b). A slight decoupling between the applied current and the potential response is observed to occur at 0.5mA/cm^2^, however, at current densities up to 0.7 mA/cm^2^ the specific area resistance is near 27 Ω·cm^2^, notably higher than that of the uncoated material 8 Ω·cm^2^. Interestingly, applying the same protocol to the cell for a second time resulted in a clearer response between the applied current and the potential (Figure S13). Notably, the cell does not appear to have shorted but rather has a stable set of specific area resistance values ranging between 23 Ω·cm^2^ and 27 Ω·cm^2^ at current densities between 0.2-0.7 mA/cm^2^.

A more careful examination of the relationship between current density and cell resistance reveals a temporary increase in cell impedance between 0.2 mA/cm^2^ and 0.3 mA/cm^2^ in both cells. The exact source of this increase remains unknown and will be the focus of future *in situ* characterization, however, we hypothesize that it could be related to the formation of a secondary interface at the electrolyte-Li boundary. In the ALD coated LPSCl, the introduction of the metal oxide, in this case MgO, would lead to differences in the composition of this interface, and might help explain the stability upon subsequent electrochemical cycling. With the goal of deconvoluting the effect of this possible reaction to the potential response of the cell, we added an additional step where the cells are cycled 0.3 mA/cm^2^ before initiating the galvanostatic protocol.

**
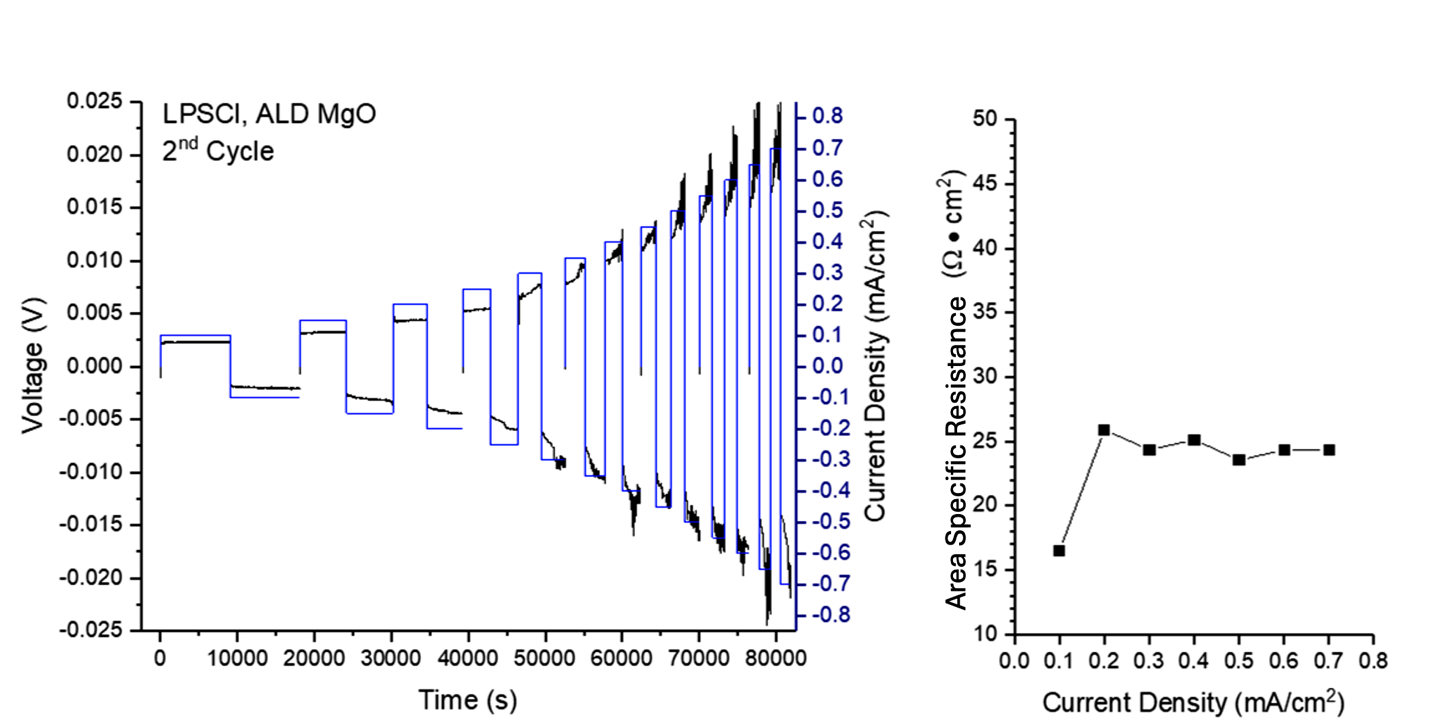
**

**Figure S13.** Electrochemical response of MgO coated LPSCl during second cycle.

**
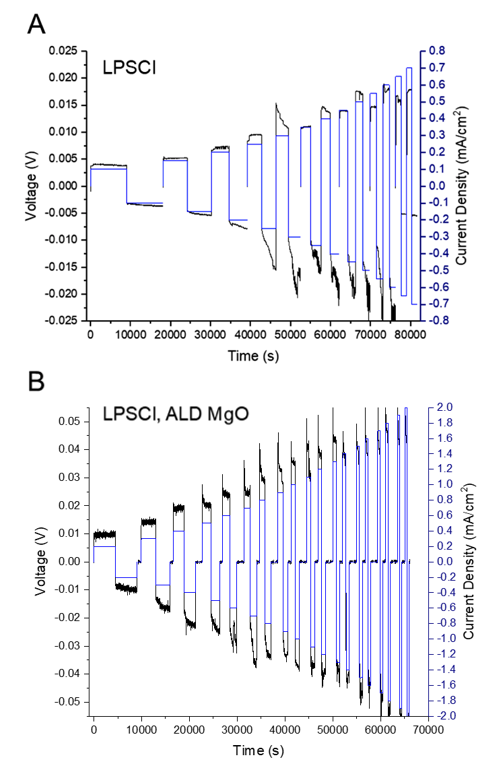
**

**Figure S14.** Critical current density measurements of (A) uncoated LPSCl and (B) MgO ALD coated LPSCl as a function of applied current densities. Data correspond to impedance results reported as Cell 2 in Figure 8.


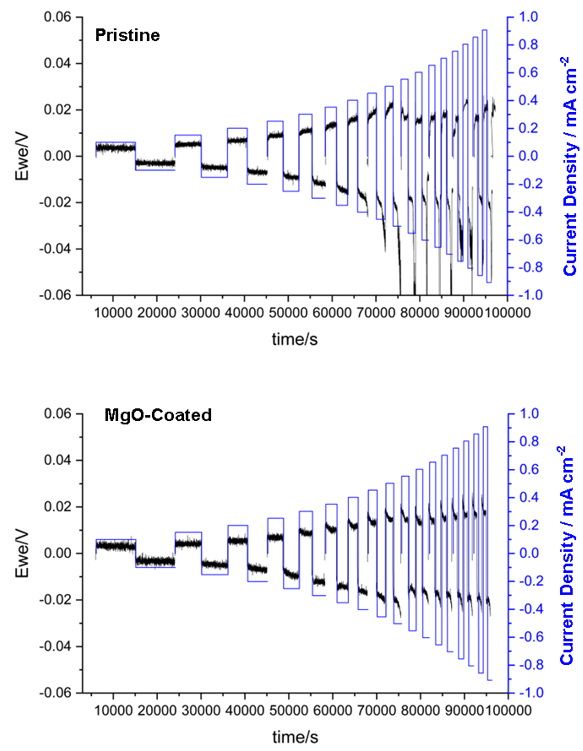


**Figure S15.** Critical current density measurements of (A) uncoated LPSCl and (B) MgO ALD coated LPSCl as a function of applied current densities. Data correspond to impedance results reported as Cell 3 in Figure 8.

**Table S10.** Conditions used for ALD on LPSCl powders. Precursor temperatures are listed separately for the metal precursor and H_2_O coreactant and RT designates room temperature. Precursor pressure corresponds to the pressure rise observed during the precursor dosing and is listed separately for the metal precursor and H_2_O coreactant. Timing is listed as t1-t2-t3-t4 where t1 and t3 are the metal precursor and H_2_O coreactant dose time, respectively, and t2 and t4 are the corresponding purge times. GPC refers to the typical growth per cycle value measured on Si(100) witness coupons by spectroscopic ellipsometry following ALD.

| **Material** | **Precursors** | **Deposition Temperature (°C)** | **Precursor Temperature (°C)** | **Precursor Pressure (Torr)** | **Timing (s)** | **GPC (Å/cycle)** |
| --- | --- | --- | --- | --- | --- | --- |
| ZrO_2_ | TDMAZr/H_2_O | 150 | 80/RT | 0.4/1.5 | 5-60-3-60 | 0.95 |
| MgO | MgCp_2_/H_2_O | 150 | 91/RT | 0.2/1.7 | 3-30-3-30 | 1.5 |
| ZnO | DEZ/H_2_O | 150 | RT/RT | 1.0/1.2 | 3-30-3-30 | 2.0 |
| Al_2_O_3_ | TMA/H_2_O | 150 | RT/RT | 0.9/1.2 | 3-30-3-30 | 1.2 |

**References**

[1] G. Deroubaix, P. Marcus, *Surface and Interface Analysis* **2016** *18,* 39–46. <https://doi.org/10.1002/sia.740180107>
